# Hospitalisations with infectious disease diagnoses in somatic healthcare between 1998 and 2019: A nationwide, register-based study in Swedish adults

**DOI:** 10.1016/j.lanepe.2022.100343

**Published:** 2022-03-24

**Authors:** Torisson Gustav, Rosenqvist Mari, Melander Olle, Resman Fredrik

**Affiliations:** a Department of Infectious Diseases, Skåne University Hospital, Malmö, Sweden; b Clinical Infection Medicine, Department of Translational Medicine, Lund University, Malmö, Sweden; c Department of Clinical Sciences Malmö, Lund University, Malmö, Sweden; d Department of Emergency and Internal Medicine, Skåne University Hospital, Malmö, Sweden.

## Abstract

**Background:**

Several studies indicate increasing hospitalisation rates for specific infectious diseases (IDs). Studies describing the entire ID spectrum are scarcer. Our aim was to describe hospital use with ID diagnoses in Swedish adults from 1998 to 2019.

**Methods:**

All four-position codes in ICD-10 were reclassified as ID or non-ID diagnoses. Using data from the National Patient Register, age-standardised hospitalisation rates and average length-of-stay (LOS) was determined for hospitalisations with ID vs non-ID diagnoses in the primary position at discharge. The 22-year study period was divided into five periods that were compared using standardised rate ratios (SRR).

**Findings:**

Annual hospitalisations with ID diagnoses increased from 115 thousand in 1998-2002 to 182 thousand in 2015-2019, for a rate increase from 17·4 to 23.0 per 1000 person-years, and a SRR (95%CI) of 1.32 (1.32-1.33). Concurrently, the hospitalisation rate with non-ID diagnoses decreased from 147 to 110, for a SRR of 0.75 (0.75-0.75). Average LOS decreased less for IDs than for non-IDs. Consequently, the proportion of hospital nights for which an ID was considered causing the hospitalisation increased from 11% to 21%. Persons aged 80+ years had the highest ID hospitalisation rate.

**Interpretation:**

The increased hospital use with ID diagnoses suggests an increasing incidence of severe IDs as well as a changing case-mix of hospitalised patients. Given the anticipated demographic change, this trend is likely to persist. Healthcare systems will need to address IDs in a comprehensive and standardised way.

**Funding:**

Governmental Funding of Research within the Clinical Sciences (ALF)

**Research in context**

**Evidence before this study**

The PubMed database was searched on October 12th 2019 using the search strategy ((Trends[MeSH Subheading]) AND (hospitalization[MeSH Terms])) AND ("infect*"[Title]). Numerous studies were found to show increasing trends in hospitalisations for separate infectious conditions, such as pneumonia or urinary tract infections. To the best of our knowledge, only three studies have aggregated ID diagnoses to describe the full ID spectrum. These include two from the US, using data from 1980 to 1994 and from 1998 to 2006, and one from New Zealand, from 1989 to 2008, showing increasing trends for ID hospitalisations. These have used different aggregates of ICD-9 and ICD-10 codes. Studies from Europe are lacking, as are studies in the last decade. In addition, previous studies have primarily focussed on hospitalisation rates, not emphasising length-of-stay into the analysis.

**Added value of this study**

This study provides a comprehensive classification of all four-position ICD-10 codes into ID or non-ID, enabling the study of an aggregate of the full ID spectrum. The classification is applied to a register with nationwide coverage of all discharge diagnoses for a 22-year period, comparing IDs to non-IDs. The results show diverging trends, where hospitalisations for IDs increase substantially with a simultaneous decrease for non-IDs. In addition, length-of-stay was included in the analysis, showing a slower decrease for IDs than non-IDs. Subsequently, the proportion of hospital use attributed to ID diagnoses increased, indicating a changing case-mix of hospitalised patients. Age stratification showed that the largest increase was seen in the oldest old. Analysis of ID subcategories suggested a change towards more complex IDs.

**Implications of all the available evidence**

The trend of increasing hospital use due to ID diagnoses is likely to persist in developed countries due to the ageing of populations. Healthcare systems will need to adapt to this development with a multifaceted approach that includes prevention and infection control, as well as the optimisation of ID management and education of healthcare professionals.

Alt-text: Unlabelled box

## Introduction

In Sweden, national projections forecast a 90% increase of persons aged over 80 years until 2050.^1^ Demographic projections from the EU and US show a similar pattern.^2^^,^^3^ Concurrently, shifting management towards outpatient care has led to a reduction in hospital beds per capita.^4^ In 2019, Sweden was the European OECD country with the fewest hospital beds per capita, with 2.1 per 1000 inhabitants, a figure that has decreased by 45% over the last 22 years. With fewer hospital beds and an ageing population, healthcare systems will need to adapt through service-planning and resource-optimisation.

Numerous studies from different countries in Europe, North America and Oceania have shown increasing hospitalisation rates for different separate ID diagnoses, including pneumonias and urinary tract infections.^5^, ^6^, ^7^, ^8^, ^9^, ^10^, ^11^, ^12^, ^13^, ^14^ Studies of the entire spectrum of IDs are scarcer. To the best of our knowledge, only three studies have aggregated ID diagnoses for a full insight of the ID spectrum .^15-17^ These include two from the US, describing the situation from 1980 to 1994 and from 1998 to 2006, and one from New Zealand, describing 1989 to 2008. European studies are lacking, as well as studies in the last decade, and studies in countries with very low hospital bed capacity. An increasing hospitalisation rate with ID diagnoses, with a decreasing overall hospital use due to fewer beds, suggest a growing proportion of hospital use due to ID diagnoses. Such a trend would imply a greater need to provide services related to IDs, including diagnostics, treatment and infection control, as well as education and training in the ID field.

Our aim was to describe hospital use with an ID diagnosis in the primary position and its progression on a national level in Swedish adults between 1998 and 2019. The overall purpose being to contribute to a discussion on resource allocation regarding ID healthcare and the upcoming demographic challenges.

## Methods

The general approach was to 1) reclassify all codes in ICD-10 as either ID or non-ID diagnoses, 2) retrieve data for adult hospitalisations from the Swedish National Inpatient Register from 1998 to 2019, and 3) describe trends, focussing on hospitalisation rates as well as hospital nights.

### Reclassification of ID diagnoses

The ICD-10 consists of 22 chapters, of which the first, chapter I - “certain infectious and parasitic diseases”, comprises only ID diagnoses.^18^ However, important ID diagnoses are found in other chapters as well, e.g. pneumonias in chapter X - “Diseases of the respiratory system”. Thus, a reclassification of diagnostic codes is needed to describe the full spectrum of ID diagnoses. A study from New Zealand has aggregated ID diagnoses, with the overall aim to describe diseases that “would be entirely or predominantly prevented if exposure to the causative organism was eliminated”.^16^ To align with our purpose, the previous study was used as a template but we further specified ID diagnoses as those considered likely to need ID-related services. In short, we defined ID diagnoses as those for which a micro-organism would have caused the admission and where treatment would primarily consist of antimicrobial therapy (or be supportive). All changes to the previous classification are specified in the supplementary appendix, along with a detailed description of definitions and the reclassification process. We reviewed all four-position ICD-10 codes and classified them as either ID or non-ID diagnoses. Reclassification was done prior to data retrieval and thus, the authors were blinded to hospitalisation data. All ID diagnoses were further reclassified into 13 major categories. Eleven of these were based on infection sites, with the remaining two being “infectious complications” and “other infections”. For infectious complications, a subcategory of device-associated infections was described. Non-IDs were categorised according to their original ICD-10 chapters.

### Codes with special concerns or that were excluded from the main analysis

Intraabdominal disorders often resist strict categorisation, as acute surgical conditions are frequently complicated by infections. We used two definitions, of which the narrower, was used as default, and the broader in a sensitivity analysis. Mental disorders (chapter V) were excluded from the main trend analysis, as psychiatric care is organised separately from somatic care in Sweden and with separate legislation, e.g., regarding discharge procedures. Hospitalisations related to pregnancies and the perinatal period (chapters XV-XVII) were also excluded from the main analysis, as trends in these would closely follow the national birth rate. The chapter regarding factors influencing health status and contact with healthcare services (chapter XXI) was also excluded as they would not represent illnesses or injuries. These chapters were included in a separate sensitivity analysis.

### Primary data source – the National Patient Register (NPR)

The Swedish edition of ICD-10, ICD-10-SE, was implemented in 1998 by the National Board of Health and Welfare (NBHW), motivating this year as the study starting point. Upon hospital discharge, a primary diagnosis is registered by the discharging medical doctor. According to national guidelines, the primary diagnosis should represent the cause of hospital admission, given the information known at discharge. Non-primary codes were not included as the registration of such codes has increased substantially over time, complicating trend analysis. All hospitals in Sweden are obliged to report to the Swedish National Patient Register (NPR), founded in 1964, which was the primary data source for this study.^19^^,^^20^ The NPR allows one primary diagnosis per discharge from a hospital department. However, during a complex hospitalisation episode with transfers between departments, several discharge diagnoses could have been registered (this was also addressed in a sensitivity analysis).

From the NPR, aggregate data was retrieved on adult hospitalisations, hospital nights and average length-of-stay (LOS), with the filters: diagnosis, year (1998 to 2019), sex, and age (in ten-year strata: 20-29, 30-39, 40-49, 50-59, 60-69, 70-79, and 80+).

### Contextual data sources

From the database at OECD, annual data on the number of hospital beds / 1000 inhabitants was retrieved.^4^ From Statistics Sweden, year-end population data, stratified by year, age and sex, including future projections, was retrieved.^1^ In addition, the annual antibiotic sales to the hospital sector, defined as DDD / TIND (defined daily doses from ATC group J01 per thousand inhabitants and day) was retrieved from the antimicrobial consumption database (ESAC-Net) at the ECDC.^21^ This measure is separated from sales to the community sector / primary care.

### Statistical methods / Endpoints

The primary unit of interest was a hospitalisation and ID vs non-ID status was defined by our classification, applied to primary discharge diagnoses from the NPR. The 22-year study period was separated into five time periods to reduce influence of specific years (such as years with severe flu seasons). The following endpoints, with in-depth methodology described in the supplementary appendix, were estimated:

**Hospitalisation rate**. This was considered a proxy for incidence of disease requiring hospitalisation. The numerator was the number of hospitalisations from the NPR, and the denominator was person-years at risk, using population data from Statistics Sweden. Rates were standardised to the 2013 European standard population to adjust for changes in population structure over time and to facilitate international comparisons.

**Average LOS**. This was considered a measure related to hospital logistics / efficiency as well as frailty / severity of disease. As aggregated data was retrieved from the NPR, only the average LOS was presented (not standard deviation, 95% CI).

**Rate of hospital nights**. This is the composite of hospitalisations * average LOS and was considered the measure most closely related to resource utilisation. For hospital nights, rates were calculated in the same way as for hospitalisations, including age-standardisation.

**Standardised rate ratio (SRR)**. This was used to compare rates within groups between study periods. For each time period, the age-standardised rate (ASR) was divided by the ASR of the first study period (1998 to 2002) that was used as the reference (SRR = 1). The interpretation is that a SRR for hospitalisation rates of 1·32 is equivalent to an increase of 32% in the number of hospitalisations for that period compared to in 1998 - 2002, adjusted for changes in the population.

**The proportion with ID diagnoses**. This was defined as the number of ID hospitalisations / hospital nights divided by the total number of hospitalisations / hospital nights in the analysis (ID diagnoses + non-ID diagnoses).

**Rate of hospital nights per hospital bed and year**. This was determined by dividing the number of hospital nights with the annual number of hospital beds, derived from the hospital bed per capita data from OECD. This was compared between periods using a rate ratio.

**Trends in antibiotics sales.** This was expressed as percental change in DDD for each period vs the first and was estimated using the data from ESAC-Net and compared graphically to the percental change in number of hospital nights with ID diagnoses. This crude form of external validation was intended as a supportive analysis.

**The future burden of ID** diagnoses. This was estimated by applying the age-specific hospitalisation rate for ID diagnoses from the latest period (2015-2019), to the age strata of demographic projections until 2050.

**Sensitivity analyses** were performed to test the robustness of our classification and data. The rationale and details are further specified in the supplementary appendix.

## Results

In all, 9187 four-position codes in the ICD-10-SE were reclassified as ID or non-ID diagnoses. The reclassification is described in detail in table S3 and S4 in the supplementary appendix. The total number of adult hospitalisations from 1998 to 2019 was about 30 million. When hospitalisations with diagnoses regarding mental disorders, normal pregnancies etc. had been excluded as described above, approximately 24 million hospitalisations (80%) remained and were included in the main analysis.

### ID diagnoses vs non-ID diagnoses

**Figure 1:**
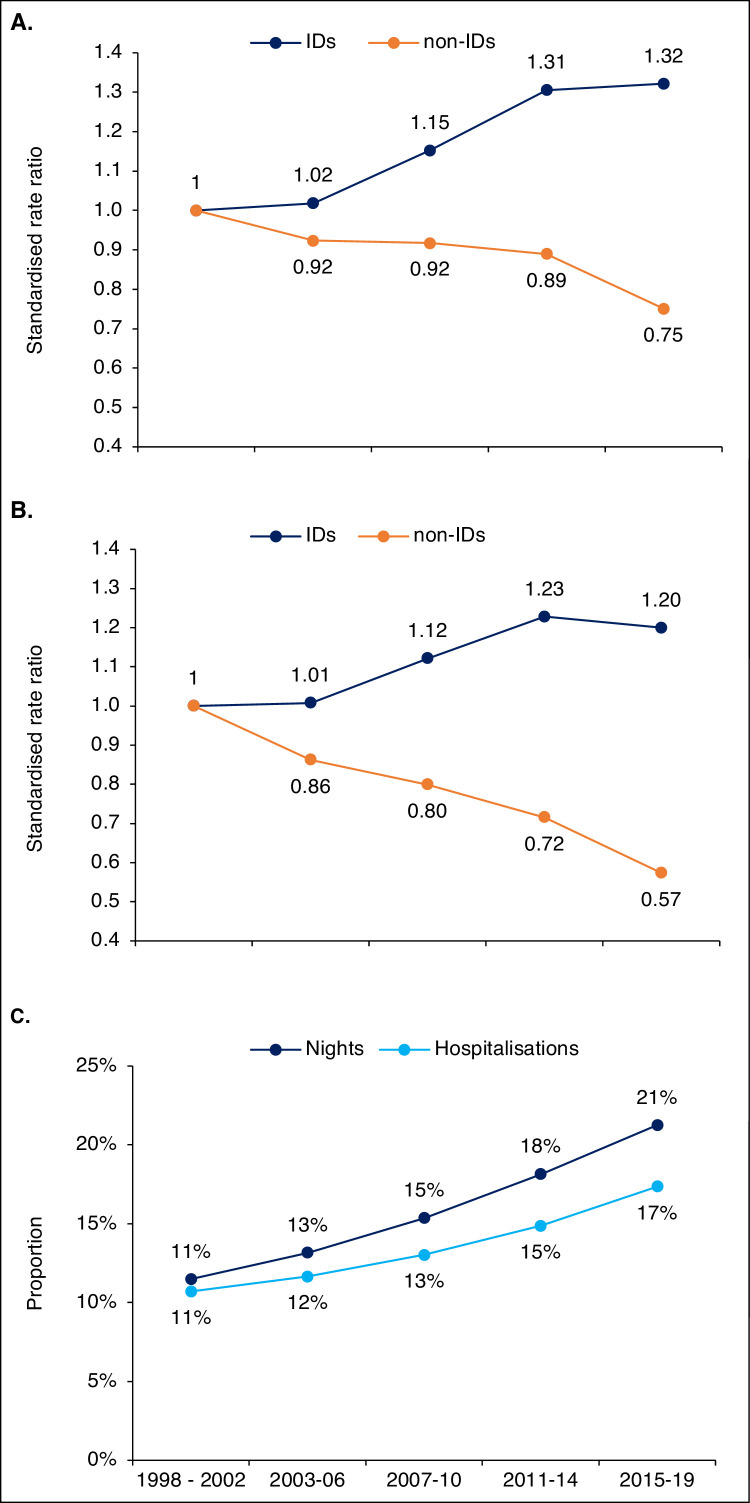
**Trends in hospitalisations and hospital nights for ID vs non-ID diagnoses.** Ratio of age-standardised rates for each period related to first period (1998 – 2002) for hospitalisations (A) and hospital nights (B) for ID and non-ID diagnoses, respectively. Trend in proportion of all hospitalisations and hospital nights with an ID diagnosis (C).

The number of hospitalisations with an ID diagnosis in the primary position at discharge increased from an annual average of 115 thousand in 1998-2002 to 182 thousand in 2015-2019, see table 1. The age-standardised hospitalisation rate increased from 17·4 to 23·0 per 1000 person-years, representing a SRR (95%CI) of 1·32 (1·32-1·33). Concurrently, hospitalisations with non-ID diagnoses decreased from an annual average of 961 thousand in 1998-2002 to 869 thousand in 2015-2019, representing an ASR decrease from 147 to 110 per 1000 person-years and a SRR (95%CI) of 0·75 (0·75-0·75). Hospitalisation trends are displayed in figure 1A. Average LOS decreased overall but were longer and decreased less for hospitalisations with ID diagnoses (from 6·4 to 5·9 nights) than for those with non-ID diagnoses (from 5·9 to 4·6 nights), see table 1. The rate of hospital nights with an ID diagnosis increased from 112 to 135 per 1000 person-years, with a SRR (95%CI) of 1·20 (1·20-1·20). Simultaneously, the rate for non-ID diagnoses decreased from 877 to 503, for a SRR (95%CI) of 0·57 (0·57-0·57), with trends shown in fig 1B. The proportion of hospitalisations and hospital nights with an ID diagnosis increased from 11% to 17% and from 11% to 21%, respectively, see figure 1C.

**Table 1:** Hospitalisations with ID and non-ID diagnoses in Sweden 1998 to 2019.

		1998 - 02	2003 – 06	2007 – 10	2011 – 14	2015 – 19	**SRR (95%CI)**
**IDs**	hospitalisations	115	121	142	168	182	
	rate	17·4	17·7	20·0	22·7	23.0	**1·32 (1·32-1·33)**
	hospital nights	738	771	892	1 023	1 070	
	rate	112	113	126	138	135	**1·20 (1·20-1·20)**
	average LOS	6·4	6·4	6·3	6·1	5·9	
**Non-IDs**	hospitalisations	961	918	950	965	869	
	rate	147	136	135	131	110	**0·75 (0·75-0·75)**
	hospital nights	5708	5112	4933	4631	3976	
	rate	877	756	700	627	503	**0·57 (0·57-0·57)**
	average LOS	5·9	5·6	5·2	4·8	4·6	

Hospitalisations and hospital nights are yearly averages (thousands), rates are events per 1000 person-years, age-standardised to the 2013 European standard population. LOS = length of stay, SRR = standardised rate ratio of the last period (2015-2019) vs the first (1998-2002). CI = confidence interval

### Stratification by age and sex

**Figure 2:**
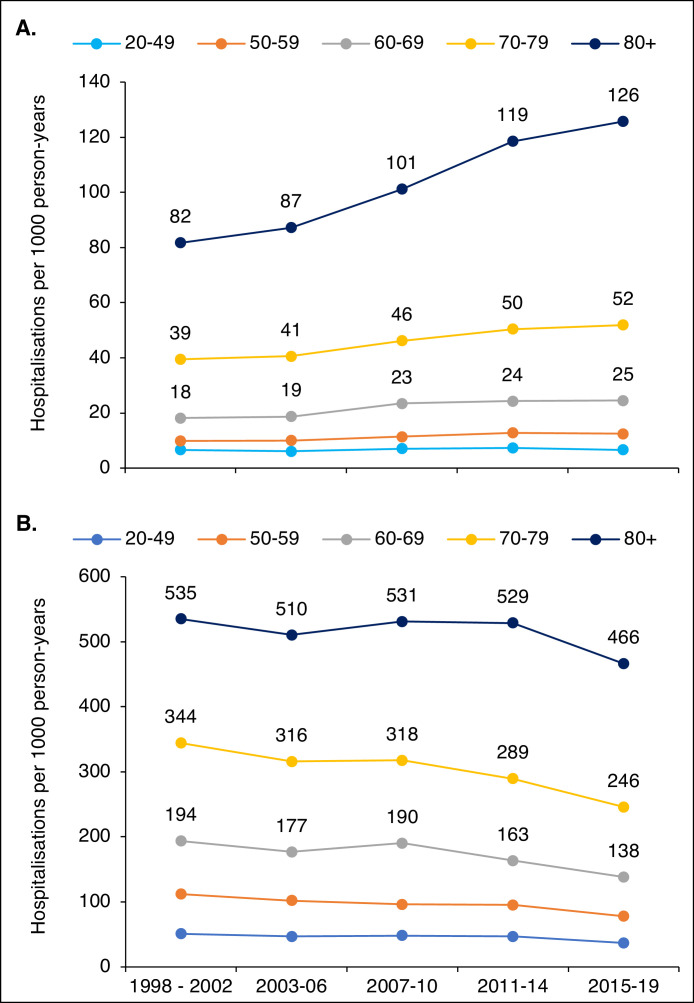
**Trends in hospitalisation rates, by age**. Age-specific hospitalisation rates for ID diagnoses (A) and non-ID diagnoses (B)

For ID diagnoses, hospitalisation rates increased in age strata over 50 years, see figure 2A. The largest proportional increase was seen in the group aged 80+ years, in whom the hospitalisation rate increased by 54%. For the groups 70-79, 60-69 and 50-59, the increases were 32%, 35% and 27% respectively. The proportion of hospitalisations with ID diagnoses found in the oldest group increased from 32% in 1998-2002 to 36% in 2015-2019. For non-ID diagnoses, hospitalisation rates decreased in all age strata, see figure 2B. The proportion of hospital nights with an ID diagnosis in the primary position at discharge increased in all age strata (from 12% to 19% for age 20-49, from 9% to 18% for age 50-59, from 10% to 19% for age 60-69, from 11% to 21% for age 70-79, and from 13% to 24% in age 80+). Trends for males and females were very similar, analyses stratified by age and sex are described in full detail in tables S9 and S10 in the supplementary appendix.

### Stratification by major diagnostic categories

**Figure 3:**
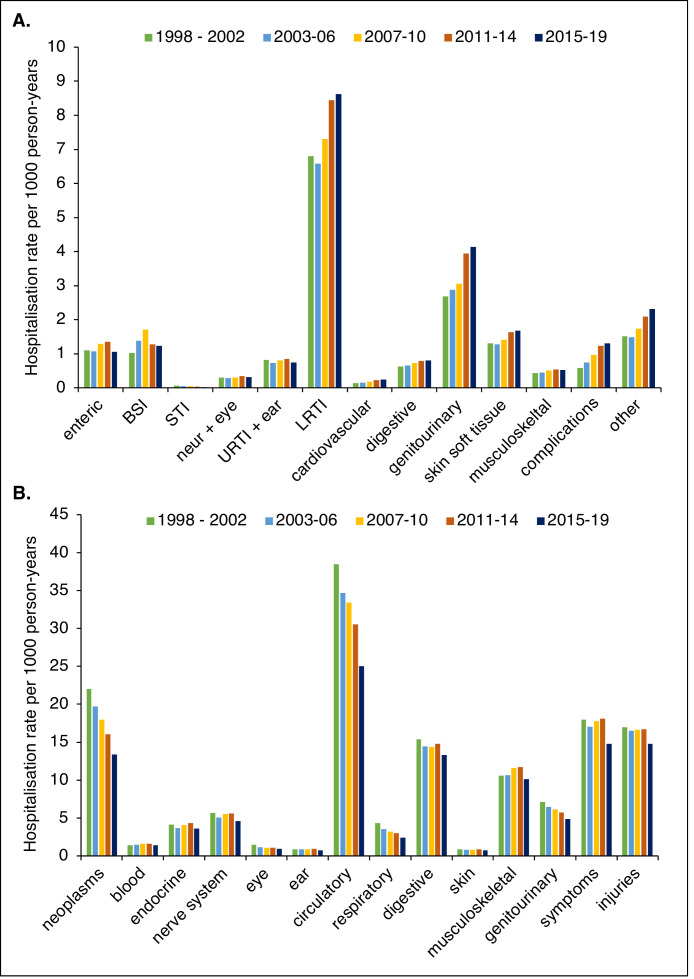
**Hospitalisation rates, by diagnostic category.** Age-standardised hospitalisation rates for major ID categories (A) and for non-ID categories, according to ICD-10 chapter (B). BSI = blood-stream infections, STI = sexually transmitted infections, URTI = upper respiratory tract infections (including ear infections), LRTI = Lower respiratory tract infections (including influenza).

Only 25% of hospitalisations with ID diagnoses were given diagnoses within ICD chapter I. The most frequent ID categories were lower respiratory tract infections (LRTIs) and urogenital infections, comprising 37% and 17% of hospitalisations with ID diagnoses, respectively. Hospitalisation rates decreased for enteric infections, sexually transmitted infections (STIs), infections of the nervous system and upper respiratory tract infections (URTIs), while increasing for the other nine ID categories, see figure 3A. The largest proportional increases in hospitalisation rates were found in infectious complications (+126%, with an increase in device-associated infections of 172%) and infections of the cardiovascular system (+83%), that also had the longest average LOS. In general, hospitalisation rates increased for ID categories with longer LOS and decreased for those with shorter LOS, see table S11 in the supplementary appendix. For non-ID diagnoses, hospitalisation rates decreased across all categories, see figure 3B. All further details on diagnostic categories are found within tables S11 and S12 in the supplementary appendix.

### Results from contextual data sources

**Figure 4:**
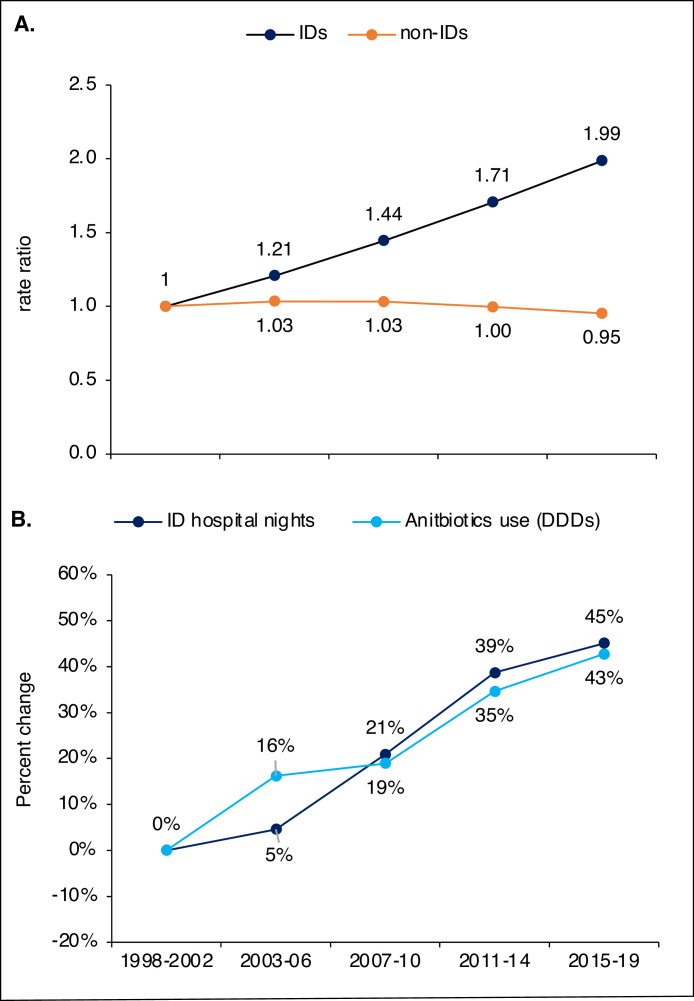
**Analysis of contextual data.** Rate ratio for the rate of hospital nights per hospital bed and year for ID and non-ID diagnoses for each period compared to 1998 - 2002 (A). Percent change from 1998 - 2002 for the number of hospital nights with ID diagnoses and antibiotics sales to the hospital sector (B).

**Figure 5:**
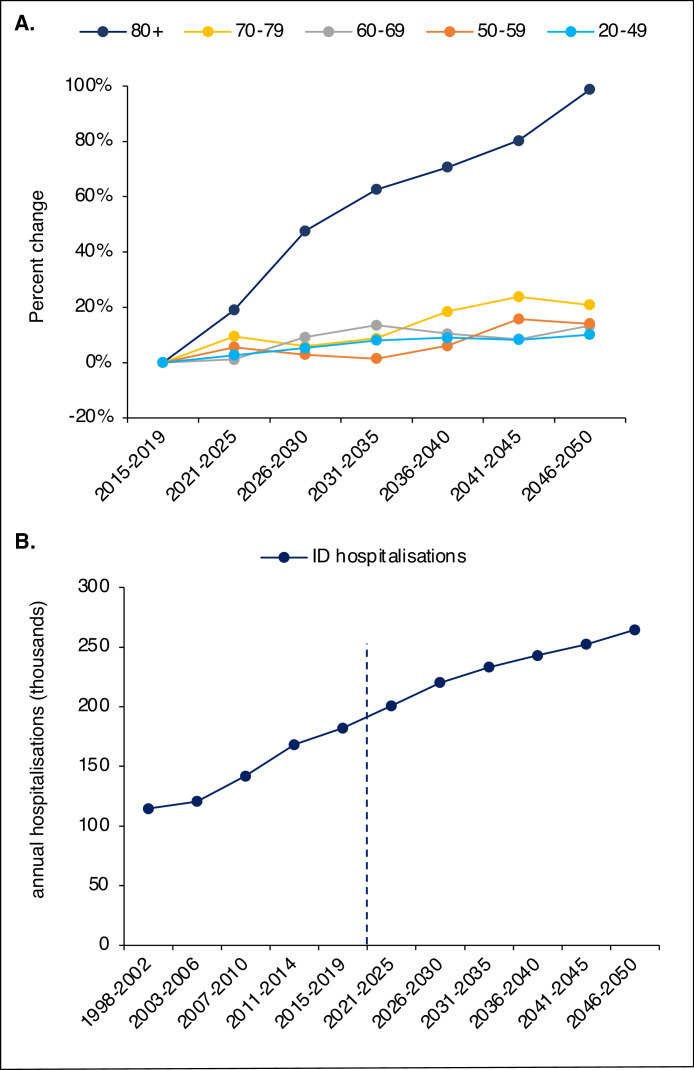
**Future projections and the impact of aging.** Projected percental change of the Swedish population by age strata up to 2050 (A). Historical and projected annual number (thousands) of hospitalisations with ID diagnoses from 1998 to 2050 (B). Projected numbers are estimated by applying age-specific rates from 2015-2019 to the projected population change.

The rate of hospital nights per year and hospital bed with ID diagnoses increased from 23·8 nights in 1998-2002 to 47·3 in 2015-2019. When rate ratios for this endpoint were plotted, the trends for ID and non-ID diagnoses were less segmented than for hospital nights alone, see figure 4A. The trend for antibiotics sales to the hospital sector displayed an increase of 43% in DDDs, from 1998-2002 to 2015-2019, compared to an increase in hospital nights of 45%, see figure 4B. The future demographic projections using data from Statistics Sweden are displayed in figure 5A. When age-specific hospitalisations rates from 2015-2019 were applied this projection, there was a projected increase from 182 thousand annual hospitalisations with an ID diagnosis in 2015-2019 to 265 thousand in 2046-2050, see figure 5B.

### Sensitivity analyses

All seven sensitivity analyses showed the same general trend, with SRR for hospitalisations ranging between 1·18 to 1·38 with ID diagnoses (main analysis 1·32) and from 0·73 to 0·82 for non-ID diagnoses (main analysis 0·75). The SRR for hospital nights ranged from 1·08 to 1·28 for ID diagnoses (main analysis 1·20) and from 0·55 to 0·67 for non-ID diagnoses (main analysis 0·57). Further details are found in table S13 in supplementary appendix.

## Discussion

We found that hospital use with ID diagnoses in the primary position increased substantially in Sweden from 1998 to 2019. This was seen in both absolute and relative terms, when compared to non-ID diagnoses. For ID diagnoses, we found an increasing hospitalisation rate and a relatively slower LOS decrease than for non-ID diagnoses. Consequently, the proportion of hospital nights for which an ID was considered having caused the admission increased from 11% in 1998-2002 to 21% in 2015-2019, suggesting a changing case-mix of hospitalised patients. This change was seen in all age strata, ruling out a changing age composition of the population as the full explanation. Analyses of ID categories suggested a shift from less complicated ID diagnoses (with shorter LOS, e.g., URTI or enteric infections) towards more complex ones, with longer LOS.

Studies from the US and New Zealand have previously reported ID hospitalisation rates of 15·4 and 18·8 per 1000 person-years in 2005 and 2004 - 2008, respectively.^15^^,^^16^ This is in line with our rates of 17·7 for the period 2003-2006, suggesting a similar incidence of severe IDs if admission thresholds were comparable. However, differences in age structures may have influenced the results as the rates have not been standardised to the same standard population. These studies also describe increasing hospitalisations rates for ID diagnoses as well as pneumonia / LRTI as the top category, comprising 34% and 30% of ID hospitalisations, respectively (compared to 37% in our study). The study from New Zealand described a larger decrease in average LOS for ID diagnoses (from 6·1 days in 1993-1998 to 4·3 in 2004-2008) than in our study (from 6·4 nights in 1998-2002 to 5·9 in 2015-2019).^16^ This could indicate a more efficient ID healthcare in New Zealand or a different case-mix, with a higher admission threshold in Swedish hospitals. As in previous studies, only 25% of hospitalisations with ID diagnosis were within chapter I, emphasising the need for a reclassification to describe the full ID spectrum.

Trends for ID and non-ID diagnoses followed a similar non-linear pattern but with different slopes (figure 1A and 1B). When the denominator was changed and annual nights per hospital bed was analysed, the segmentation was far less pronounced with a linear increase for ID-diagnoses (figure 4A). This may represent a gradual rise in the underlying ID incidence, but other factors could have impacted the results. First, changes in the admission threshold could have affected trends in ID hospitalisations. We consider a lowered hospital threshold for IDs unlikely, given the overall decrease in bed capacity and the suggested shift towards more complicated IDs. Second, as we relied on ICD-codes to designate IDs, changes in coding or coding practices could have imitated trends. All code conversions were scrutinised without indications of affecting the main results. In addition, we screened common ID diagnoses for signs of changes in coding practices, as described in the supplementary appendix. Nevertheless, coding practices may differ over time and across regions and changes unknown to the authors could have influenced the results. Third, many ID diagnoses are based on criteria rather than histopathological findings, making validity lower than for cancer diagnoses etc. Thus, improved methods to screen for and diagnose IDs may also have affected the results. However, the supportive analysis of antibiotics sales to the hospital sector strengthened the suggestion of an actual increase in the incidence of severe IDs. Increasing antibiotics sales to the hospital sector with a concurrent decrease in hospital beds also support the idea of a changing case-mix in hospitalised patients.

The mechanism underlying the increase in hospitalisation rates for ID diagnoses was not studied. Sweden has the lowest national bed capacity among OECD countries, signalling that the transition to outpatient care has come a long way. We hypothesise that organisational and medical advances in other fields may have contributed. For example, the rate of surgical procedures increased by 17% during the period, primarily in older persons.^22^ More complex surgeries in older patients, performed in the outpatient setting, could raise complications rate and thus hospitalisations with ID diagnoses.^23^ In our study, the hospitalisation rate with infectious complication diagnoses more than doubled, and the increase in device-associated infections was even higher. In addition, cancer incidence rates increased during the study period.^22^ Concurrently, the hospitalisation rate with a discharge diagnosis within chapter II – “neoplasms” decreased, indicating a shift towards outpatient management. However, if infectious complications occur at a constant rate, hospitalisation rates with ID diagnoses would increase.^24^ Furthermore, the national prescription rate of immunosuppressants in the national prescribed drug register has doubled from 2006 to 2019.^25^ Anti-inflammatory drugs have revolutionised treatment for certain disorders, e.g. rheumatoid arthritis, for which hospitalisations in the NPR have dropped dramatically. Still, these drugs entail a heightened risk of infectious complications needing hospitalisation.^26^ These are examples of medical and organisational improvements that could affect the case-mix from both aspects, by reducing hospitalisation rates for non-ID diagnoses while simultaneously increasing them for ID diagnoses. In addition, policy changes related to IDs, including immunisation policies may have influenced hospitalisations rates.

Our study has several limitations. First, only aggregate data, without individual identifiers, was used, limiting the analysis to variables in the NPR. Thus, hospitalisation data could not be linked to socioeconomic data, underlying health conditions and other public health factors. The clinical validity of ICD diagnoses does have uncertainties, although a study of a broad range of diagnoses within the NPR has demonstrated good validity.^19^ Third, our classification of ID diagnoses is based on clinical experience and inherently subjective, and previous studies have used slightly different classifications. We acknowledge this and have tested the stability of the classification in several sensitivity analyses. Fourth, only hospital data was used; data from primary care could have provided further insight but was not available within the NPR. Fifth, results from Sweden may not be generalisable to other healthcare systems, with regard to the organisation of ID healthcare, the low bed capacity and antimicrobial resistance levels. Sixth, including non-primary diagnoses could have provided information regarding infections occurring during the hospitalisation, including healthcare-associated and nosocomial infections. Without this data, the full impact of IDs may have been underestimated. However, as in previous studies, there was a considerable increase in coding frequency of non-primary codes during the study period, complicating any trend analysis.^16^^,^^17^^,^^27^

The rationale of the present study included forecasting the impact of the upcoming demographic changes. When age-specific rates of 2015-2019 were applied to the projected population of 2046 - 2050, the projected number of hospitalisations with ID diagnoses increased >40% (figure 5B). This prognosis, isolating the effect of projected ageing, must be interpreted with great caution as it assumes the unlikely scenario that rates will stabilise at the 2015 - 2019 level. Improvements in ID healthcare could come to lower hospitalisation rates. On the other hand, rising levels of bacterial resistance could force a lowered admission threshold, increasing proportion of IDs to require in-hospital treatment.^28^ There is uncertainty in population projections as well and we acknowledge that this is a crude form of forecasting, meant to roughly estimate the impact of projected demographic change to hospital utilisation for IDs.

Regarding implications, our finding that 25% of ID hospitalisations were found in chapter I of ICD-10, underline the necessity of manually aggregating ID diagnoses to appreciate the full ID spectrum. Our classification may serve as a framework for researchers in countries with nationwide registers. Future studies linking registers with individual level data could overcome limitations of this study and provide insight into possible explanations. Further studies should also investigate trends in important subcategories, including healthcare-associated infections. Regarding clinical implications, the high and increasing incidence of LRTI in elderly necessitates an improved coverage of vaccines, e.g. influenza immunisation, that currently has a coverage in targeted risk groups < 40% in Europe.^29^ The relatively slower LOS decrease for ID diagnoses suggest that hospital logistics could be improved for IDs, including outpatient intravenous treatment, also in long-term care facilities. From a policy-making perspective, the Covid-19 pandemic has highlighted the need for surveillance as well as flexibility in short-term resource-allocation. The increasing proportion of hospital use with ID diagnoses in the present study indicates also a long-term need of ID-related services, to ensure microbiological diagnostic capacity as well as adequate ID training and education for healthcare professionals.

## Conclusion

We found an increasing proportion of hospital use with ID diagnoses in the primary position during 1998 to 2019, suggesting an increasing incidence of severe IDs as well as a changing case-mix of hospitalised patients. Given the anticipated demographic change, this trend is likely to persist. After the Covid-19 pandemic, healthcare systems will need to address IDs in a multifaceted and strategic way.

## Contributors

GT and FR conceived the study. GT, MR, OM and FR developed the study design. GT performed data collection, analysis and interpretation. The underlying dataset and analysis was verified by FR. The manuscript was drafted by GT and critically reviewed by MR, OM and FR. All authors have approved the final version of the manuscript.

### Role of the funding source

This study was supported by the Governmental Funding of Clinical Research within the National Health Services (ALF). The funder of the study had no role in the design, data collection, analyses or write-up of this report. The authors had full access to all study data and final responsibility for the decision to submit for publication.

### Ethics statement

Ethical approval for this study was waived by the Swedish Ethical Review Authority as the study was based on aggregate data (2020-04845).

### Data availability statement

The data used in this study is accessible from the National Board of Health and Welfare (https://www.socialstyrelsen.se/en/statistics-and-data/). Aggregate data on three-position ICD-10 codes can be found in an online database and four-position codes can be requested.

## Declaration of interests

All authors have completed the ICMJE uniform disclosure form and declare: no support from any organisation for the submitted work; no financial relationships with any organisations that might have an interest in the submitted work in the previous three years; no other relationships or activities that could appear to have influenced the submitted work.

## Appendix Supplementary materials

Image, application 1
