# Supplementary material for: Hospitalisations with infectious disease diagnoses in somatic healthcare between 1998 and 2019: A nationwide, register-based study in Swedish adults
Source: Lancet Reg Health Eur. 2022 Mar 24;16:100343. doi: 10.1016/j.lanepe.2022.100343 (PMC8960944; doi:10.1016/j.lanepe.2022.100343)
Supplement: Supplementary file 1 [file mmc1.pdf]

SUPPLEMENTARY APPENDIX

Hospitalisations with infectious disease diagnoses in somatic healthcare between 1998 and 2019: A nationwide, register-based study in Swedish adults

Torisson G, Rosenqvist M, Melander O, Resman F

INTRODUCTION ... 3

Population projections ... 3
Hospital bed capacity ... 3
Studies of trends for ID hospitalisation rates ... 3

METHODS - SETTING ... 4

The healthcare system in Sweden ... 4
The International Classification of Diseases and Related Health Problems, 10th edition, Swedish edition (ICD-10-SE) ... 4
The Swedish reimbursement system ... 4

METHODS - DATA SOURCES ... 5

The National Patient Register ... 5
The Statistics Database from Statistics Sweden ... 5
Antibiotics data from ESAC-NET ... 5
OECD health statistics ... 5

METHODS - RECLASSIFICATION PROCESS ... 6

Previous studies defining IDs ... 6
Criteria for defining ID diagnoses ... 6
Process to identify ID diagnoses ... 6
Diagnoses that were removed from ID classification from Baker et al. ... 7
Diagnoses that were added to the ID classification by Baker et al. ... 9
Intraabdominal infections ... 11
Categorisation of ID and non-ID diagnoses ... 11
Changes in coding and coding practices ... 12
Changes in coding ... 12
Changes in coding practices ... 13
Hospitalisations that were excluded from trend analysis ... 14

METHODS – STATISTICAL ANALYSIS ... 15

Example: hospitalisations with ID diagnoses in 1998-2002 and 2015-2019. ... 15
Crude rates with 95% confidence intervals ... 15
Age-standardised rates (ASR) with 95% confidence intervals ... 16
Standardised rate ratios (SRR) with 95% confidence intervals ... 16
Software used ... 17
Analyses including contextual data ... 17
Adjusting for hospital bed capacity ... 17
Comparison with antibiotic sales to the hospital sector ... 17
Extending to population projections ... 18
Sensitivity analyses ... 18

RESULTS ... 19

ID vs non-ID diagnoses ... 19
Stratification by age and sex ... 19
Major diagnostic categories ... 19

Rate of hospital nights per hospital bed and year 19
Sensitivity analyses 19

FIGURES 20

Figure S1. Population projection for Sweden 20
Figure S2. Population projection for the European Union 20
Figure S3. Population projection for the USA 21
Figure S4. Trends in hospital bed capacity in Sweden 1998 to 2019 22
Figure S5. Trends in hospital bed capacity vs hospital use in Sweden 1998 to 2019 22
Figure S6. Yearly hospitalisation rates for IDs and non-ID diagnoses 23
Figure S7. Yearly rates for hospital nights for IDs and non-ID diagnoses 23
Figure S8. Hospitalisation rate by major ID category and age 1998 to 2019 24
Figure S9. Standardised rate ratio for hospital nights for IDs vs major non-ID categories 24
Figure S10. Rate ratio for the rate of hospital nights per bed and year for IDs and non-IDs 25

TABLES 26

Table S1. Studies of hospitalisation rates with ID diagnoses 26
Table S2. Coding issues for common ID diagnoses 27
Table S3. ID diagnoses, by major ID category 28
Table S4. Diagnoses classified as non-IDs, by ICD-10 chapter 29
Table S5. The most frequent four-position ID diagnoses overall 30
Table S6. The most frequent codes in each ID major category 31
Table S7. Hospitalisations included and excluded from analysis 32
Table S8. Hospitalisations and hospital nights, by year 33
Table S9. Hospitalisations with ID diagnoses, by age and sex 34
Table S10. Hospitalisations with non-ID diagnoses, by age and sex 35
Table S11. Hospitalisations with ID diagnoses, by major category 36
Table S12. Hospitalisations with non-ID diagnoses, by ICD-10-SE chapter 38
Table S13 Sensitivity analyses 40

REFERENCES 42

Introduction

Population projections

In Sweden, national projections forecast a 90% increase of persons aged over 80 years until 2050.[1] This is virtually the only age group projected to increase. Concurrently, the overall population is projected to increase by 15%. The same pattern is seen in projections from the EU as well as the US, see figures S1 – S3.[2,3]

Hospital bed capacity

In 2019, Sweden was the European OECD country with the fewest hospital beds per capita, with 2.07 per 100 000 population. Over the last 22 years, this number has decreased by 45%, with a sharp decrease between 1998 and 2003, see fig S4. This trend is reflected in hospital use; If the trends in hospital nights and hospital beds are standardised to percental change from 1998 levels and plotted, they are visibly correlated, see figure S5.

Studies of trends for ID hospitalisation rates

Numerous studies from different countries in Europe, North America and Oceania have shown a increasing hospitalisation rate for different separate ID diagnoses.[4-18] To the best of our knowledge, only three studies have aggregated ID diagnoses for a full insight of the ID spectrum.[19-21] These include two from the US, describing the situation from 1980 to 1994 and from 1998 to 2006, and one from New Zealand, describing 1989 to 2008. See table S1. European studies are lacking, as well as studies in the last decade, and studies in countries with very low bed capacity. An increasing hospitalisation rate, with a decreasing overall hospital use due to fewer beds, suggest a growing proportion of hospital use for ID diagnoses.

Methods - Setting

The healthcare system in Sweden

Healthcare expenditure in Sweden amounts to 11.0% of the national GDP (2017).[22] The public health care system is decentralised and controlled both financially and operationally by 21 regional county councils. Somatic healthcare is divided into primary care and hospital-based specialised care. Home care, rehabilitation and long-term care are controlled by municipalities, a smaller local government entity of which there are 290 in Sweden. When a patient is no longer in need of medical hospital care, the responsibility for the person's care is transferred to the municipality through a discharge planning. Typically, this would mean that a patient is discharged when there is no further need for daily monitoring, intravenous treatments, oxygen etc. However, there may still be need for rehabilitation, physiotherapy and assistance with activities of daily living. There are 70 county hospitals in Sweden, as well as seven larger university hospitals.

The International Classification of Diseases and Related Health Problems, 10th edition, Swedish edition (ICD-10-SE)

The first Swedish translation of ICD-10, called "Klassifikation av sjukdomar och hälsoproblem 97" (KSH97), was introduced by the National Board of Health and Welfare (NBHW) in 1997.[23] The system was employed nationally from 1998 onwards. In 2011, the name was updated to ICD-10-SE. In the first four positions, the ICD-10-SE is identical to the WHO version, with a few discrepancies. National additions have been made to the Swedish edition of ICD-10 however, added in order to distinguish certain diagnoses more clearly. These 2800 national additions are designated with a letter in the fifth position, e.g., E116D "type 2 diabetes mellitus, with diabetic foot ulcer". Some diagnoses come in "asterisk-dagger" pairs, where two primary diagnoses could be used to describe both aetiology and locale of disease, for example the asterisk code G01* "Meningitis in bacterial diseases classified elsewhere" could be accompanied by a dagger code such as A321+ "Listerial meningitis and meningoencephalitis".

Upon hospital discharge, a principal ICD-10-SE diagnosis is decided by the physician responsible for the patient´s discharge. According to national guidelines, this diagnosis should represent the cause of admission, given the information known at discharge. The principal diagnosis controls the designation to one of 24 Major Diagnostic Categories (MDCs). These diagnostic categories are further subclassified, by the principal diagnosis, into one of approximately 500 separate DRG (diagnose-related groups).

The Swedish reimbursement system

In Sweden, the reimbursement system is decentralised and the 21 county councils decide on their own reimbursement models for healthcare providers, including hospitals. For hospital care, the reimbursement models are different between county councils and use either global budgets or a mix of global budgets, case-based or performance-based reimbursement.[22] Per-case payments are to different degrees related to a DRG weight, that is decided annually by each county council and is based on actual healthcare costs.

Methods - Data sources

The National Patient Register

The National Patient Register was founded by the National Board of Health and Welfare in 1964.[24] All hospitals in Sweden are obliged to report to the register, and for hospitalised patients the coverage is above 99%. The register contains data regarding age, sex, diagnoses, admission and discharge dates, etc. A main ICD-10-SE diagnosis is listed upon discharge for almost all patients (missing for 0.5% – 0.9%).[25] Aggregated data with three ICD-10-SE positions is available to anyone from the NBHW website, and four-position codes are available on request.[26] If both diagnoses of an asterisk-dagger pair have been registered as main diagnoses, the dagger code is prioritised as the main diagnosis in the National Patient Register and the asterisk code is removed. In a few cases however, a single asterisk code is erroneously registered without a dagger code and then that code is retained. The validity of the register has been studied with regard to several specific diagnoses, with chart review and death certificates as comparators. With these measures, a diagnosis in the register had a positive predictive value of 85-95%.[25]

A main discharge ICD-10 diagnosis will be registered for all patients discharged from a Swedish hospital department. The main diagnosis should be the one causing the symptom(s) for which the patient was admitted to the department, given the diagnostic workup performed to that point.[27] This means that if the patient is transferred between departments during a complex hospitalisation episode, several main diagnoses could be registered. The register also contains secondary diagnoses. Traditionally, Swedish hospitals have registered few secondary diagnoses, compared to those in other countries. However, the number of secondary diagnoses per hospitalisation has increased substantially during the last decades.[28]

The Statistics Database from Statistics Sweden

Statistics Sweden is a governmental organisation responsible for coordinating the official statistics of Sweden. Statistics Sweden is legally obliged to provide official statistics for information, investigation, and research that is objective and publicly available. The online statistics database contains statistics within several areas, including demographic data regarding the population of Sweden. The official demographic projections are also provided by the online statistics database.[1]

Antibiotics data from ESAC-NET

The European Centre for Disease Control (ECDC) collects data on antimicrobials used in community care and within the hospital sector through the European Surveillance of Antibiotic Consumption Network (ESAC-Net).[29] The data is accessible at the interactive Antimicrobial consumption database. Antibiotic consumption is expressed as the annual number of defined daily doses (DDDs) per 1000 inhabitants per day from the group J01 "systemic antibacterials" in the ATC (Anatomical-Therapeutical Chemical) classification system. The population denominator used is also supplied.

OECD health statistics

The Organisation for Economic Co-operation and Development tracks a large number of healthcare indicators, published yearly in the *OECD Health at a glance* series. The indicators, including hospital beds per capita, are also available at the OECD health statistics database.[30] Hospital beds per capita is expressed as beds / 1000 inhabitants on an annual basis and include curative (acute) care beds, psychiatric care beds, rehabilitive care beds, long-term care beds and other beds in hospitals.

Methods - Reclassification process

Previous studies defining IDs

The main issue with studying IDs as a group in registers is that they are distributed over many different ICD chapters. Although the first chapter – "Certain infectious and parasitic diseases" comprises only IDs, there are many other chapters that also contain IDs, e.g., respiratory tract infections in Chapter X – "Diseases of the respiratory system", or urinary tract infections in Chapter XIV – "Diseases of the genitourinary system". Previous studies have shown that only about 20-25% of IDs are found in Chapter I.[19,31] Therefore, an aggregation that includes diagnostic codes from other chapters has to be used in order to fully define IDs.

We studied previous efforts to group IDs from diagnostic codes thoroughly. Notable studies include the 1996 study by Pinner et al. in which ICD-9 codes were used in order to determine mortality trends in the US.[21] This grouping has also been used in other studies in order to determine trends in hospitalisations.[20] When scrutinising this definition, we determined that it requires an update. Obviously, it needs to translate to ICD-10. In addition, some ID categories are lacking, notably within skin and soft tissue infections, connective tissue infections, and febrile neutropenia. Baker et al. updated the original coding scheme by Pinner et al. for use in New Zealand to study trends in ID hospitalisations from 1989 to 2008.[19] As the change from ICD-9 to ICD-10 was effectuated during the study period, a translation into ICD-10 was performed. The authors' intention was to describe diseases that "would be entirely or predominantly prevented if exposure to the causative organism was eliminated". This intention, focussing on aetiology, led to a broader inclusion of diseases in different categories in which IDs play a role in the development of disease, such as cervical cancer, hepatocellular carcinoma, etc. Previous studies with different aims have thus resulted in different classifications of IDs.

Criteria for defining ID diagnoses

Our aim was somewhat different than that of Baker et al.; i.e., to describe trends with the overall purpose of contributing to a discussion regarding resource allocation within the healthcare system, with regard to services related to IDs. Therefore, we further specified ID diagnoses as diagnoses for which:

- An infection with a micro-organism would likely be responsible for causing the current hospital admission, rather than being a risk factor for subsequent disease. For example, human papillomavirus is involved in pathogenesis of cervical cancer, but services related to IDs would primarily not be needed in a hospitalisation with a principal diagnosis of cervical cancer.

AND

- Treatment should typically consist of antimicrobial drugs and/or drainage, aiming to eliminate the causative organism, if possible, alternatively be supportive. Therefore, diagnoses such as reactive arthritis or Guillain-Barré syndrome, where treatment would be anti-inflammatory, were not considered ID diagnoses.

Process to identify ID diagnoses

We used the previous grouping schemes as starting points for our classification. As Baker et al. was the most comprehensive, contemporary, and inclusive study using ICD-10 codes it was used as a template from which all differences are commented below. To maximise generalisability and reproducibility, only four-position codes were included; national codes were omitted. We first studied all ICD-10 codes that were within the previous classification by Baker et al. and determined whether they fulfilled our definition as well, based on a clinical perspective. Any removal of codes from that definition is commented on below. All other four-position ICD-10-SE codes were subsequently scrutinised and classified as either an ID or a non-ID. Any addition to the previous classification by Baker et al. is also commented on below. The codes were selected from a complete list of 9187 ICD-10-SE four-position codes without beforehand knowledge of the number of hospitalisations or trends.

Diagnoses that were removed from ID classification from Baker et al.

Most of the diagnoses that were removed could be generally classified within certain categories:

1. Long-term effects from chronic infection, where the infection would not be considered the main problem during hospitalisation and where treatment is complex and generally not antimicrobial. Examples of diagnoses in this group include neoplasms, such as cervical or hepatocellular cancer.

2. Secondary, inflammatory effects that are primarily post-infectious and where the infection is no longer the main problem. Treatment would typically focus on anti-inflammatory effects. Examples in this group include Guillain-Barré syndrome or reactive arthritis.

3. Diagnoses with a multifactorial pathogenesis with an unclear role of infection, where the majority of cases are presumed to be non-infectious. These are often chronic conditions where antibiotic treatment is generally not warranted. Examples from this category include chronic sinusitis or nonsuppurative otitis media.

The following diagnoses were removed from the previous classification:

- C11 – **Malignant neoplasm of nasopharynx**. Epstein-Barr virus is associated in aetiology but would not be considered the main problem.[32] Oncological treatment. Classified as non-ID. Approx. 130 cases / year.
- C16 – **Malignant neoplasm of stomach**. Helicobacter pylori is involved in aetiology but not the main problem during a hospitalisation.[33] Oncological/surgical treatment. Classified as non-ID. Approx. 2200 cases / year.
- C210 and C211 – **Malignant neoplasm of anus and anal canal**. Human Papilloma Virus is involved in aetiology but not the main problem. Treatment is surgical and oncological.[34] Classified as non-ID. Approx. 290 cases / year.
- C220 – **Liver cell carcinoma**. Liver disease in general and hepatitis B in particular is a risk factor.[35] Treatment of manifest disease is primarily oncological. Classified as non-ID. Approx. 670 cases / year.
- C46 – **Kaposi sarcoma**. HIV and Human herpesvirus 8 (HHV8) are main contributing factors. However, there is a code also for HIV disease resulting in Kaposi sarcoma (B21). If C46 is the primary diagnosis for a hospitalisation we considered it to represent a need for oncological treatment rather than HAART.[36] Classified as non-ID. Approx. 10 cases / year.
- C53 – **Malignant neoplasm of cervix uteri**. Human papilloma virus involved in aetiology but no target for treatment, which is oncological.[37] Classified as non-ID. Approx. 1500 cases / year.
- D002 – **Carcinoma in situ of stomach**. Classified as non-ID. See C16 for rationale. Approx. 5 cases / year.
- D013 – **Carcinoma in situ of anus and anal canal**. Classified as non-ID. See C210 for rationale. Approx. 5 cases / year.
- D06 – **Carcinoma in situ of cervix uteri.** Classified as non-ID. See C53 for rationale. Approx. 310 cases / year.
- E033 – **Post-infectious hypothyroidism**. Post-infectious by definition. Treatment consists of thyroid hormone. Classified as a non-ID. <5 cases / year.
- F024* - **Dementia in human immunodeficiency** disease (B22.0+). We excluded diagnoses from Chapter V. Asterisk code. <5 cases / year.
- F071 – **Post-encephalitic syndrome**. Post-infectious by definition. We excluded diagnoses from Chapter V. Approx. 6 cases / year.
- G030 – **Non-pyogenic meningitis**. In the Swedish version of ICD-10, this has been translated to the equivalent of "non-infectious" meningitis. Therefore, classified as a non-ID. Approx. 10 cases / year.
- G048 – **Other encephalitis, myelitis, and encephalomyelitis**. The only description of this code is post-infectious encephalitis and encephalomyelitis NOS. Therefore, it was considered primarily post-infectious. Treated with anti-inflammatory drugs. Classified as a non-ID. Approx. 45 cases / year.
- G09 – **Sequelae of inflammatory diseases in the central nervous system**. The description states that this code should be used one year or more after a diagnosis in G00 – G08. It was a considered a late effect, where the initial infection is no longer the main problem. Classified as a non-ID. Approx. 5 cases / year.
- G610 – **Guillain Barré syndrome**. This was considered a post-infectious disorder. Classified as a non-ID[38]. Approx. 260 cases / year.

- H65 – **Nonsuppurative otitis media**. This is another multifactorial condition where the role of infection is unclear.[39] Guidelines recommend against treating with antibiotics.[40] Considered a non-ID. Approx. 60 cases / year.
- H680 – **Eustachian salpingitis.** This condition may follow a rhinosinusitis, but the infection is not the main problem. Treatment consists of nasal decongestants and steroids.[41] Classified as a non-ID. <5 cases / year.
- I00 – I02 – **Acute rheumatic fever.** Although the triggering infection is treated with antibiotics, the autoimmune response to streptococcal infection is considered the main problem.[42] Classified as a non-ID. Approx. 10 cases / year.
- I05 – I09 – **Chronic rheumatic heart disease**. This was considered a late effect of previous infection.[43] Classified as a non-ID. Approx. 250 cases / year.
- I716 – **Thoracoabdominal aneurysm, without mention of rupture.** The rationale for including this code was not commented on by Baker et al.[19] Infections such as salmonella may be involved in the pathogenesis of aortic aneurysm, but we considered this a non-ID. Approx. 90 cases / year.
- I880 – **Nonspecific mesenteric lymphadenitis**. This was considered primarily as an inflammatory disease where treatment is anti-inflammatory.[44] Classified as a non-ID. Approx. 80 cases / year.
- J32 – **Chronic sinusitis**. We considered chronic sinusitis to be primarily of inflammatory aetiology.[45] Classified as a non-ID. Approx. 500 cases / year.
- J37 – **Chronic laryngitis**. We considered chronic laryngitis to be multifactorial, where infections play a small part, if any.[46] Classified as a non-ID. Approx. 90 cases / year.
- J40 – J42 **Chronic bronchitis**. We considered this a part of the umbrella term of COPD and not an ID per se.[47] There are codes signifying when an acute exacerbation or pneumonia is present. Classified as a non-ID. Approx. 600 cases / year.
- J47 – **Bronchiectasis**. Another multifactorial disease where infections may play a part in the development of disease, along with many other factors.[48] Infections may also be the cause of exacerbations resulting in hospitalisations. In contrast to COPD however, there are no codes specifying exacerbations. Classified as a non-ID. Approx. 130 cases / year.
- J988 – **Other specified respiratory disorders.** We considered this code too unspecific and a non-ID. Approx. 130 cases / year.
- K02 – **Dental Caries**, K050 – **Acute gingivitis**, K052 – **Acute Periodontitis** and K053 – **Chronic periodontitis**. These are relatively mild dental conditions in which the oral bacterial flora play a role in the pathogenesis, which is a long-standing process.[49] Treatment with antibiotics is not generally recommended. Of the oral infections, we included only those with osteitis and / or abscesses.[50] The above codes were thus considered non-IDs. Approx. 110 cases / year.
- K293 – K295 **chronic gastritis.** This was considered a chronic inflammatory disease where Helicobacter pylori plays a part in the pathogenesis but would not be considered the main problem.[51] Classified as a non-ID. Approx. 130 cases / year.
- K35 – K37. **Appendicitis**. The role of microbes in the pathogenesis of appendicitis is unclear. Most guidelines emphasize a “surgery first” approach, although an “antibiotics first” strategy could be used in specific patients.[52] Appendicitis was primarily considered a non-ID. However, acute appendicitis (K35) was included in a sensitivity analysis, see separate section on intraabdominal infections below. Approx. 7800 cases / year.
- K528 – **Other specified noninfective gastroenteritis and colitis**. Specified as noninfective, we considered this a non-ID. Approx. 250 cases / year.
- K529 - **Noninfective gastroenteritis and colitis, unspecified.** Specified as noninfective, we considered this a non-ID. See also section on code changes below. Approx. 1900 cases / year.
- K632 – **Fistula of intestine**. Although this may be caused by an infection as well as complicated by an infection, we considered this code too unspecific.[53] Treatment of the fistula is mainly surgical. Classified as non-ID. Approx. 210 cases / year.
- K908 – **Other intestinal malabsorption**. This code includes Whipple’s disease, which is a very rare infectious disease. But the code was considered too unspecific and includes for example gall salt malabsorption. Classified as a non-ID. Approx. 10 cases / year.
- M021 – **Arthritis after dysentery**. This was considered primarily an immune-mediated condition, where infection is primarily predisposing.[54] Treatment is anti-inflammatory. Classified as a non-ID. <5 cases / year.
- M023 – **Reiter’s syndrome**. Considered post-infectious and thus a non-ID. Approx. 7 cases / year. The small numbers of M021 and M023 may be due to a predominant use of the code M029 **Reactive arthropathy NOS**. In total, the M02 - **Reactive arthropathy** code comprises approx. 350 cases / year. This general code was not included as an ID by Baker et al. or by us.

- M03* - **Post-infectious and reactive arthropathies in diseases classified elsewhere**. By definition post-infectious. Classified as non-ID. < 5 cases / year.
- M896 – **Osteopathy after polio**. Considered post-infectious. Non-ID. <5 case / year.
- N00 – N05 **Glomerulonephritis**. This condition is also multifactorial where an initial infection may play a role. However, the main problem is most likely a late, immune-mediated effect.[55] Accordingly, glomerulonephritis was considered possibly post-infectious but not primarily an ID. Approx. 120 cases / year.
- N351 – **Post-infective urethral stricture, not elsewhere classified**. By definition post-infective. Classified as a non-ID. Approx. 8 cases / year.
- N411 – **Chronic prostatitis.** The pathogenesis of this condition is also multifactorial, with an unclear role of bacteria.[56] In the absence of concurrent urinary tract infection, antibiotic treatment is not recommended.[56,57] Considered a non-ID. Approx. 15 cases / year.
- N87 – **Dysplasia of cervix uteri**. In analogy with cervical cancer C53, this was not considered an ID. Approx. 300 cases / year.
- R11 – **Nausea and vomiting**. This code represents an unspecific symptom that may be caused by several different conditions, some of which are infectious. If caused by an infection such as gastroenteritis, there are several codes that should primarily be used for this. In conclusion, the code was considered unspecific and unlikely to represent an ID in the majority of cases. Approx. 3000 cases / year.
- S1013, S1083, S1093, S2013, S2033, S2043, S2083, S3083, S3093, S4083, S5083, S6083, S7083, S8083, S9083, T009, T0903, T1108, T1303, T1403. Baker et al. included codes for superficial injuries to designate "other skin infections". These used five-position codes, such as S1013, S1083, etc., that are not used in ICD-10-SE. These were considered unspecific and thus non-IDs. When four-position codes were used, such as S101, S108, etc., there were approx. 560 cases / year combined.
- R761, R762, T36, T37, T485, T487, T490, T495, T496, T499, T788, T789. These are codes used to describe adverse reactions to ID treatment with antibiotics etc. We did not consider these IDs. Approx. 70 cases / year.
- T633 and T634 - **Toxic effects of contact with venomous animals and other arthropods,** respectively. These were considered non-IDs. Approx. 90 cases / year.
- T881 – **Other complications following immunization**. This is specified as rash following immunization. Considered a non-ID. Approx. 5 cases / year.
- T887 – **Unspecified adverse effect of drug or medicament.** This diagnosis was considered too unspecific and not an ID. Approx. 650 cases / year.
- T8901 and T8902. These codes have no equivalent in ICD-10-SE and were thus excluded from both the ID group and the non-ID group.

Diagnoses that were added to the ID classification by Baker et al.

These diagnoses were within the following categories:

1. Asterisk codes. These should not be the main diagnoses, but if they were, they were included. Typically, these are conditions of which there are very few cases.

2. Diagnoses where the definition of the diagnosis as well as the contribution of infection are somewhat unclear but where the majority of cases could be presumed to be infectious, and antibiotics would typically be recommended. Since all diagnoses had to be designated as IDs or non-IDs, these were considered IDs. This group includes aspiration pneumonia and COPD with acute exacerbation.

3. Diagnoses where the definition and extent of infection is unclear but that represent an important clinical entity requiring ID services that is not represented by any other codes. Examples in this group include febrile neutropenia and fever of unknown origin.

The following diagnoses were added to the previous classification:

- D70 – **Agranulocytosis**. Febrile neutropenia is a major infectious complication of cancer treatment. None of the previous ID classifications' aggregating ICD codes have attempted to include febrile neutropenia.[19,21] Although the name "agranulocytosis" is rather unspecific, we deemed that if this diagnosis is the primary diagnosis during a hospitalisation, there was most likely a suspected infection. There is no other ICD code to designate febrile neutropenia and the ICD code for agranulocytosis has

previously been used with this purpose in register-based studies.[58,59] Generally, this code would be used primarily when the source and aetiology of infection are unknown, otherwise organ specific (e.g. pneumonia) or bacteria specific (e.g. septicaemia due to other gram-negative organisms) would be prioritised. Sometimes it is also used in addition to the code R50.8 “other specified fever”. Thus, D70 alone could underestimate the incidence of the clinical entity of febrile neutropenia. While Sweden has no formal guidelines on how to code febrile neutropenia, the most prominent online resource for clinical support, internetmedicin.se, only recommends the use of the code D70.[60] Classified as an ID. Approx. 620 cases / year.

- D733 – **Abscess of spleen**. This was considered an ID and was added. Approx. 10 cases / year.
- E060 – **Suppurative thyroiditis**. This was considered an acute infection, typically caused by bacteria.[61] Approx. 6 cases / year.
- H162 – **Keratoconjunctivitis**, H168 – **Other keratitis** and H169 – **Keratitis unspecified**. Baker et al. only includes the code H160 – corneal ulcer. H162 represents the epidemic form of keratoconjunctivitis by e.g. adenovirus. In register-based studies on incidence of infectious keratitis the ICD codes H168 and H169 are also used.[62] Classified as an ID. Approx. 190 cases / year.
- H610 – **Perichondritis of the external ear.** This is a bacterial infection of cartilage that is treated with antibiotics and, in case of abscess, drainage.[63] Classified as an ID. Approx. 30 cases / year.
- J441 **Chronic obstructive pulmonary disease, with acute exacerbation.** In ICD-10-SE, the COPD syndrome is coded using four codes: 1) J440 – **COPD with acute lower respiratory infection**, 2) J441 – **COPD with acute exacerbation**, 3) J448 – **Other specified COPD** and 4) J449 – **COPD, NOS**. It is believed that 70-80% of exacerbations are caused by bacterial or viral infections.[64] We considered exacerbations severe enough to result in hospitalisation more likely to be caused by infections than not.[65] Therefore both J440 and J441 were classified as ID diagnoses. Approx. 9000 cases / year.
- J69 - **Pneumonitis due to solids and liquids** – This code is unspecific and somewhat misleading, as it is the only code that designates the aspiration syndrome. The code has been applied in register-based studies to determine prevalence and mortality in aspiration pneumonia. We consider this code unspecific but with a majority of cases being infectious and being treated with antibiotics. Classified as an ID.[66-68] Approx. 1200 cases / year.
- K047 – **Periapical abscess without sinus**. This diagnosis is paired with K046 – Periapical abscess with sinus, which was included by Baker et al. It is a deep dental infection that is treated with drainage and antibiotics.[69] Considered an ID. Approx. 110 cases / year.
- K102 - **Inflammatory conditions of jaws**. Includes osteitis of jawbone. Considered an ID. Approx. 50 cases / year.
- L303 – **Infectious dermatitis**. Considered an ID. 30 cases / year.
- M726 – **Necrotizing fasciitis**. ID. Code added 2005, see discussion below on code changes. Average 110 cases / year.
- M86 – **Osteomyelitis**. This diagnosis was included in the some of the tables in the appendix of the article by Baker et al. (in Table 1. Coding issues) but is missing from others (Table 4. ID Diagnostic groups and associated ICD codes). It was included as an ID in our study. Approx. 830 cases / year.
- N159 **Renal tubulointerstitial disease, unspecified**. The description for this code in the ICD classification is “Infection of Kidney NOS”. It was thus included as an ID. Approx. 10 cases / year.
- N309 - **Cystitis NOS**. The code is unspecific, but we considered that the majority of these would be infectious as there are other codes specifying chronic cystitis, irradiation cystitis as well as other cystitis. Approx. 1300 cases / year.
- N340 – **Urethral abscess**. Considered an ID. Approx. 10 cases / year.
- R50 – **Fever of unknown origin.** This syndrome is multifaceted and differential diagnosis is primarily within the categories of infectious diseases, inflammatory diseases and malignancy. Even though IDs would not typically be the cause in the majority of cases, excluding infectious disease is often the first priority due to their urgency. Thus, patients requiring hospitalisation would often be given empirical antibiotics. The codes of R50 includes R502, **Drug-induced fever**, R508, **Other specified fever** and R509 **Fever, unspecified**. R508 is sometimes used to designate fever in neutropenia. Of these codes, R508 and R509 were included as IDs. See also note on coding change below. Approx. 3400 cases / year.
- R572 – **Septic shock** and R650 and R651 – **Systemic inflammatory response syndrome (SIRS) caused by infection with and without organic dysfunction,** respectively. These are codes that should not be used as a primary code but as a secondary code. Therefore, any hospitalisations with these as primary codes would be erroneously coded. Designating them as non-IDs does not make sense however, and they were included as IDs. Approx. 50 / year.

- **Asterisk codes that were added, all with < 5 cases / year on average:**
  G940, H061, H192, H320, I320, I321, I520, I521, I681, I980, I981, K67, K931, M490, M491, M492, M493, M730, M731, M900, M901, M902, N080, N160, N290, N291, N330, N770, N771

Intraabdominal infections

Intraabdominal disorders are difficult to classify, as many acute surgical conditions are complicated by infection, e.g., hernia with gangrene, diverticulosis with perforation, cholecystitis, etc. Many surgical emergencies are treated with antibiotics as well as surgery for source control, where antibiotics may be prophylactic or a part of sepsis treatment. The definitions by both Pinner et al. and Baker et al. are similar regarding intraabdominal infections and include appendicitis, anorectal abscesses, intestinal abscesses, peritonitis, liver abscesses, and cholangitis as IDs. This is however not entirely intuitive, for example regarding the inclusion of appendicitis but not diverticulitis.

Our approach was to make two definitions, one **narrower**, including:
- Abscess of anal and rectal regions – K61
- Abscess of intestine – K630
- Acute peritonitis – K650
- Liver abscess – K750
- Cholangitis – K830

And one **broader**, including conditions that would be sterile inflammation at first but often complicated by subsequent bacterial infection:
- Ulcers with perforation – K251, K252, K255, K256, K261, K262, K265, K266, K271, K272, K275, K276, K281, K282, K285, K286
- Acute appendicitis – K35
- Hernias with gangrene – K401, K404, K411, K414, K421, K431, K434, K437, K441, K451, K461
- Acute mesenteric ischemia – K550
- Ileus and intestinal obstruction – K56
- Diverticular disease of intestine – K57
- Calculus of gallbladder with cholecystitis – K800, K801
- Calculus of bile duct with cholangitis – K803
- Acute cholecystitis – K804, K810, K819

To avoid overstating IDs, the narrower definition was used as the default. A sensitivity analysis was performed using the broader definition.

Categorisation of ID and non-ID diagnoses

All ID diagnoses were categorised into 13 separate subcategories designating major ID categories. This is a crude categorisation, based on infection site, adapted from the study by Baker et al. The categories were enteric infections, blood-stream infections (BSI), infections of the neurological system + eye, upper respiratory tract infections + ear (URTI), lower respiratory tract infections + influenza (LRTI), cardiovascular infections, infections of the digestive system including the liver, skin and soft tissue infections, infections of the musculoskeletal system, urogenital infections, infectious complications, and other infections. Non-ID diagnoses were categorised according to their original ICD chapters, e.g., chapter X - “diseases of the respiratory system”.

Changes in coding and coding practices

Apart from a difference in actual disease incidence, there may be other factors influencing trends in principal diagnoses. These include changes in diagnostic codes or changes in coding practice (e.g., due to improved diagnostics or changed preferences).

Changes in coding

The ICD-10 was used during the full study period. All code conversions are available at the NBHW website. Code conversions regarding ID diagnoses that occurred during the study period were analysed to determine if there were any alterations leading to significant switches, primarily between the ID and the non-ID group. These included the following:

A09 – Diarrhoea of presumed infectious origin

Before 2007, the code A09 was used for "Diarrhoea of presumed infectious origin". In 2007, A09 was divided into A090 – "Other gastroenteritis and colitis of infectious origin" and A099 – "Gastroenteritis and colitis of unspecified origin". These codes also relate to the codes of K528 "Other specified noninfective gastroenteritis and colitis" and K529 – "Non-infective gastroenteritis and colitis, unspecified". The coding change apparently led to a major switch of 3000 patients from K529 to A099 in New Zealand in 2008, thus moving substantial amount of patients from a non-ID category to an ID category.[19] We saw no such dramatic switch in 2007 in our material when A099 was introduced. The number of cases with K528 + K529 saw a steady decline prior to 2007 as well as after 2007. Therefore, we do not consider this coding change as major or in need of any further action.

M726 – Necrotising fasciitis

This code was added in 2005. Simultaneously, the code M725 – "Fasciitis not otherwise classified" was discontinued. However, the code M725 had only 30 hospitalisations on average annually, while M726 started off with 86 hospitalisations during its first year and rose to 120 in the second. We did not see a reciprocal decrease in streptococcal sepsis or any other obviously related disorder. This may represent a significant impact of the code change; however, the absolute numbers are quite low. Except for M725, it is not obvious which code might have been used to designate necrotising fasciitis prior to 2005. We considered it probable that the codes that were used would be found in the ID group. Therefore, the only action was to use M725 in the period 1998 – 2004 and M726 from 2005 – 2018.

R50 – Pyrexia of unknown origin

New subdivisions of R50 were added in 2006. These included R502 – "Drug-induced fever" and R508 – "Other specified fever". R509 – "Fever, unspecified" remained. We included R50 as an ID in our material, following the rationale described above. Since R502 is not an ID, these were considered non-ID from 2006. Naturally, there might have been drug-induced fever patients in the R50 group before 2006. However, after the initiation of this code, R502 only represented 1.8% of R50 hospitalisations overall. Therefore, no imputation or other action was taken.

R572 – Septic shock, R650 and R651 – SIRS of infectious origin with and without organ dysfunction.

The code R572 – "Septic shock" was added in 2010. However, guidelines state that this code should only be used as a supplementary code to describe the severity of infection and not as the primary diagnosis. The code was erroneously given as a primary diagnosis in approx. 50 hospitalisations yearly after 2010. Our estimation is that these patients most likely were given a (correct) diagnosis that would be found within the ID group prior to the introduction of R572 in 2010. Thus, we do not believe that the introduction of this code during the study period has had a substantial impact on the overall ID incidence numbers. The same rationale applies to R650 – "Systemic inflammatory response syndrome (SIRS) of infectious origin without organ dysfunction" and R651 – "SIRS of infectious origin with organ dysfunction". These codes were added in 2007 and should not be used as the primary diagnosis code either. They were erroneously given in approx. 40 hospitalisations yearly after 2007.

Changes in coding practices

There are several situations in which coding practices may change. These include improved diagnostics and specification, e.g., there has been a sharp increase during the period for the diagnosis A08.1 “acute gastroenteropathy due to Norwalk factor”, probably due to increased availability of rapid diagnostic tests. Simultaneously, there has been a reciprocal decline of the diagnosis A08.4 “viral intestinal infection, unspecified”. Other reasons may include changed coding guidelines or informal and / or local changes.

To screen for coding changes, all four-position ID diagnoses that contributed with more than 1% to the total number of ID diagnosis hospitalisations during the period were screened graphically for issues including sharp increases/decreases on single years or very large overall changes, reflecting a change in policy during the study period. If such issues were found, explanations were sought. The issues found are presented here and in table S2:

Improved diagnostics in pneumonia in 2005

The national guidelines for pneumonia management changed in 2005, to include nasopharyngeal culture as a routine.[70] After this change of guidelines, the rates for diagnoses J139 “Pneumonia due to streptococcus pneumoniae”, J149 “Pneumonia due to Haemophilus Influenzae” and J159 “Bacterial pneumonia” increased markedly, possibly due to more bacteria being found in nasopharyngeal cultures. Concurrently, the proportion of diagnoses that are microbiologically unspecified, as J180 “Broncopneumonia” and J181 “Lobar pneumonia” decreased and J189 “pneumonia, unspecified” was almost unchanged. These changes did not change ID vs non-ID status for any diagnoses, neither was ID category affected.

Changed guidelines for sepsis coding in 2011

Updated recommendations for sepsis coding were published in 2011.[71] The recommendations specified that organ-specific diagnoses (e.g. N10 “acute tubulo-interstitial nephritis”) should be prioritised as the principal diagnosis and that codes within A40-A41 (e.g. A41.5 “sepsis due to other gram-negative organisms”) should only be used as the principal diagnosis when the infectious focus was unknown. This was followed by a sharp decrease in principal diagnoses within A40-A41, seemingly accompanied with a reciprocal increase in codes of urinary tract infections, and to some extent pneumonias. This change did not alter ID vs non-ID status, but analysis of ID categories was affected.

Seemingly reciprocal changes

There were a few reciprocal changes in adjacent codes where we could find no other explanation than change of preference. For example, the code J44.1 “COPD with acute exacerbation, unspecified” increased with a reciprocal decrease of J44.0 “COPD with acute lower respiratory infection” and J209 “acute bronchitis” When these codes were combined, a more linear trend appeared. There was also a large increase in rate for the code N39.0 “Urinary tract infection, site not specified”. Concurrently, there was a recriprocal decrease in rate for acute cystitis N30.0 and N30.9.

These are all examples of how changes in coding and coding practices have led to significant changes in trends for specific four-position ICD-10 codes but not to a switch between ID and non-ID diagnosis status.

Hospitalisations that were excluded from trend analysis

1. Diagnoses in Chapter V – "Mental and Behavioural disorders" were excluded, as psychiatric care is separately organised and regulated by a different legislation. For example, when a patient is no longer considered in need of hospital care but will be transferred to community care. In psychiatry, the community services had three months' time to initiate community care up until 2003, when the time was decreased to 30 days. For somatic care, the same time frame was five days until 2018, then three days. Thus, discharge routines and length-of-stay would be related to such changes and difficult to compare. In addition, psychiatric care in Sweden is typically organised separately from somatic care, with separate emergency departments, separate wards etc.

2. Diagnoses from Chapter XV – "Pregnancy, childbirth and puerperium", were also excluded. This category is dominated by normal deliveries, and trends in hospitalisations, even for infectious complications, would be more closely related to the national birth rate than any other underlying changes.

3. Diagnoses within Chapter XVI – "Certain conditions originating in the perinatal period", and Chapter XVII – "Congenital malformations, deformations and chromosomal abnormalities" were also excluded, since they would be considered erroneously given to adults.

4. Diagnoses from chapter XXII – "Factors influencing health status and contact with health services" were also excluded from trend analysis as they would typically not represent illness or injury.

Chapters XV, XVI, XVII, and XXII have also been excluded from previous work seeking to define IDs vs non-IDs. The characteristics of these chapters are described in sensitivity analysis.

Methods – Statistical analysis

Here we present the statistical methodology and examples of calculations of crude rates, age-standardised rates (ASR) and standardised rate ratios (SRR), with 95% confidence intervals.

Example: hospitalisations with ID diagnoses in 1998-2002 and 2015-2019.

The table below shows data regarding ID hospitalisations for the periods 1998 to 2002 and 2015 to 2019.

		1998 - 2002					2015-2019				
age	std	events	pyrs	rate	for asr	for var	events	pyrs	rate	for asr	for var
20-29	12 000	33 592	5 532 915	0.00607	73	0.158	38 812	6 707 959	0.00578	69	0.124
30-39	13 500	41 255	6 310 424	0.00654	88	0.189	40 832	6 473 569	0.00630	85	0.178
40-49	14 000	41 978	5 871 052	0.00715	100	0.239	50 411	6 512 056	0.00774	108	0.233
50-59	13 500	59 877	6 122 945	0.00978	132	0.291	78 528	6 327 354	0.01241	168	0.357
60-69	11 500	74 777	4 133 137	0.01809	208	0.579	137 747	5 631 180	0.02446	281	0.574
70-79	9 000	137 851	3 502 834	0.03935	354	0.910	240 116	4 639 707	0.05175	466	0.903
80+	5 000	183 994	2 255 414	0.08158	408	0.904	323 826	2 579 653	0.12553	628	1.217
SUM	78 500	573 324	33 728 721		1363	3.270	910 272	38 871 478		1805	3.587

age = age in 10-year strata
std = standard population (in this case 2013 European standard population)
events = number of hospitalisations with an ID diagnosis in the age strata during the period.
pyrs = person-years at risk, sum of year-end population of the years in the period, within each stratum
rate = events / pyrs
for asr = rate * standard population – this parameter is used to calculate ASR
for var = (standard population^2 * rate) / pyrs – this parameter is used to calculate variance for ASR and SRR

Crude rates with 95% confidence intervals

Crude rates were not presented. However, the methodology is used to estimate age-specific rates, as presented in figure 2 in the main text. Confidence intervals were generally not presented in figures, as they were too narrow to be seen. Crude rates with 95% confidence interval were calculated:

$$crude\ rate = \frac{\sum_{i=1}^{k} events_i}{\sum_{i=1}^{k} pyrs_i}$$

$$95L = \frac{\chi^2_{(\alpha/2,\ 2*events)}}{2} / pyrs$$

$$95U = \frac{\chi^2_{(1-(\alpha/2),\ 2*(events+1))}}{2} / pyrs$$

Where $events_i$ is the number of events at the ith stratum, α the desired level of confidence (e.g., 0.05). χ^2 represents the quantile of the χ^2 distribution for the desired probability p and degrees of freedom (The corresponding value can be retrieved by using the CHISQ.INV (p, df) function in Microsoft Excel or the function `stats::pchisq(p, df)` in R software.

Example: hospitalisations with ID diagnoses in 1998 – 2002 and 2015 – 2019
Crude rate$_{1998\text{-}2002}$ = 573324 / 33728721 = 0.016998
95L$_{1998\text{-}2002}$ = (CHISQ.INV (0.025, 2 * 573324) / 2) / 33728721 = (1143682 / 2)/ 33728721 = 0.01695
95U$_{1998\text{-}2002}$ = (CHISQ.INV (0.975, 2 * (573324 + 1)) / 2 / 33728721 = (1149620 / 2) / 33728721 = 0.01704

Crude rate$_{2015\text{-}2019}$ = 910272 / 38871478 = 0.023417
95L$_{2015\text{-}2019}$ = ((CHISQ.INV (0.025, 2 * 910272) / 2) / 38871478= 1816806 / 2 / 38871478 = 0.02337
95U$_{2015\text{-}2019}$= ((CHISQ.INV (0.975, 2 * (910272 + 1)) / 2 / 38871478= (1824288 / 2) / 38871478 = 0.02347

So, when multiplied by 1000 and rounded to two decimals, the crude hospitalisation rates with 95% CI for ID diagnoses during 1998 to 2002 and 2015 to 2019 were 17.00 (16.95 to 17.04) and 23.42 (23.37 to 23.47) per 1000 person-years, respectively

Age-standardised rates (ASR) with 95% confidence intervals

Age-standardised rates are weighted mean event rates, where the weights are decided by a standard population, in this case the 2013 European standard population. The formula for standardised rates is:

$$ASR = \frac{\sum_{i=1}^{k} stdpop_i * rate_i}{\sum_{i=1}^{k} stdpop_i}$$

$$variance = \frac{\sum_{i=1}^{k}(stdpop_i^2 * rate_i \,/\, pyrs_i)}{\left(\sum_{i=1}^{k} stdpop_i\right)^2}$$

$$CI = ASR \pm Z_{\alpha/2} * \sqrt{variance}$$

Example: hospitalisations with ID diagnoses in 1998 – 2002 and 2015 – 2019

$ASR_{1998\text{-}2002}$ = 1363 / 78500 = 0.017368
$Variance_{1998\text{-}2002}$ = 3.270 / 78500 ^ 2 = 0.0000000005306
$95L_{1998\text{-}2002}$ = 0.017368 - 1.96 * √ (0.0000000005306) = 0.017368 – 1.96 * 0.00002304 = 0.017323
$95U_{1998\text{-}2002}$ = 0.017368 + 1.96 * √(0.0000000005306) = 0.017368 + 1.96 * 0.00002304 = 0.017413

$ASR_{2015\text{-}2019}$ = 1805 / 78500 = 0.022997
$Variance_{2015\text{-}2019}$ = 3.270 / 78500^2 = 0.0000000005821
$95L_{2015\text{-}2019}$ = 0.022997 - 1.96 * √ (0.0000000005821) = 0.022997 – 1.96 * 0.00002413 = 0.022949
$95U_{2015\text{-}2019}$ 0.022997 + 1.96 * √(0.0000000005821) = 0.022997 + 1.96 * 0.00002413 = 0.023044

Thus, the ASR (95% CI) for hospitalisation with an ID diagnosis in 1998 to 2002 and 2015 to 2019 was estimated to 17.37 (17.32 – 17.41) and 23.00 (22.95 to 23.04) per 1000 person-years, respectively

Standardised rate ratios (SRR) with 95% confidence intervals

To evaluate the difference between the rates of two time periods, the standardised rate ratio (SRR) was estimated. The formula for SRR is:

$$SRR = \frac{ASR_{period\ 2}}{ASR_{period\ 1}}$$

$$var(\log(SRR)) = \frac{variance_{period\ 1}}{\left(ASR_{period\ 1}\right)^2} + \frac{variance_{period\ 2}}{\left(ASR_{\ period\ 2}\right)^2}$$

$$CI = exp\left[\log(SRR) \pm z_{\alpha/2} * \sqrt{var(\log(SRR))}\right]$$

Example: hospitalisations with ID diagnoses in 1998 – 2002 and 2015 – 2019

$$SRR = \frac{ASR_{2015-2019}}{ASR_{1998-2002}} = 0.022997/0.017358 = 1.3241$$

$$var(\log(SRR)) = \frac{variance_{1998-2002}}{(ASR_{1998-2002})^2} + \frac{variance_{2015-2019}}{(ASR_{2015-2019})^2} = \frac{0.0000000005306}{0.01737^2} + \frac{0.0000000005821}{0.02300^2} = 0.000002860$$

$95L = exp\left[\log(1.3241) - 1.96 * \sqrt{0.000002860}\right] = exp[\log(1.3241) - 0.003314] = exp[0.2807 - 0.003314] = 1.3197$

$95U = exp\left[\log(1.3241) + 1.96 * \sqrt{0.000002860}\right] = exp[\log(1.3241) + 0.003314] = exp[0.2807 + 0.003314] = 1.3285$

Thus, the SRR (95% CI) of hospitalisation rate with an ID diagnosis between periods 2015-2019 and 1998-2002 was 1.32 (1.32 to 1.33). This is equivalent to an increase in hospitalisation rate of 32%, adjusted for changing population structure over time.

Software used

These calculations were performed step-by-step using Microsoft Excel. There may always be errors in such estimations and therefore our estimates were double-checked using the statistical software R and the packages `popEpi` and `heaven`. There were some small discrepancies at the fourth decimal position, but otherwise results were largely similar as seen in the example table below:

	Manual	popEPI::rate	heaven:dsr (Wald CI)
Crude rate	16.99809	16.99809	16.99809
95L	16.95412	16.95415	16.95412
95U	17.04215	17.04215	17.04215
ASR	17.36779	17.36779	17.36779
95L	17.32264	17.32270	17.32264
95U	17.41293	17.41299	17.41293
SRR last vs first	1.324099	1.324	1.324099
95L	1.319718	1.324	1.319718
95U	1.328495	1.324	1.328495

Analyses including contextual data

We performed three additional analyses including contextual data:

Adjusting for hospital bed capacity

We wanted to explore the relation between hospital use and national bed capacity. The rationale for this was to evaluate the impact of hospital bed reduction on our findings. As bed capacity is related to resource utilisation, we considered hospital nights the appropriate outcome for the situation.

1. First, the approximate number of hospital beds per year was estimated by multiplying the OECD rate by the year-end population (e.g., in 1998 the total population of Sweden was 8 854 322 and the OECD rate 3.75 per 1000 inhabitants. Thus, we estimated the number of hospital beds to be 3.75 * 8 854 322 / 1000 = 33 204 in 1998.

2. Then, we divided the number of hospital nights per period with the total hospital beds per year to obtain the rate of *hospital nights per year and hospital bed*. For ID diagnoses in 1998 to 2002 this rate would be 3 687 788 / 154 909 = 23.8 nights / year. In 2015 to 2019, the number would be 5 350 304 / 113 163 = 47.3.

3. To compare time periods, we estimated the rate ratios of the rate of hospital nights per year and hospital bed. To compare the rates for ID diagnoses from 2015-2019 to 1998-2002 we would divide 47.3 / 23.8 = 1.99. This is what is presented in figure 4A in the main document.

4. Interpretation: For each hospital bed, the number of nights where the patient had an ID diagnosis in the primary position at discharge doubled during the study period. Naturally, this measure is related to the proportion of hospital nights with ID diagnoses.

Comparison with antibiotic sales to the hospital sector

As a crude form of validation with data from another source, independent on coding practices etc. we wanted to compare our results with antibiotics use. Again, hospital nights was considered the most appropriate outcome to compare, as antibiotics sales were expressed as defined *daily* doses.

1. As antibiotics sales were defined as DDD / TIND, we first multiplied the measure with 365.25 to obtain the rate of DDD / 1000 person-years.

2. This could have been compared to hospital nights / 1000 person-years but neither numerators (DDD sales included to pediatric, obstetric departments etc.) nor denominators were not the same (for DDD entire population, for hospital nights only those aged ≥ 20 years). Therefore, we extrapolated the DDD /

1000 person-years into the number of DDDs by multiplying the rate by the population denominator from ECDC / 1000.

3. The number of DDDs was then compared to the number of hospital nights, using percental change between each period and the first. There are several pitfalls in this analysis, and it was not used for any detailed conclusions but rather as a supportive analysis of the main results. If the trend for antibiotics sale to the hospital sector would have been decreasing, there would of course be concern of the validity of ID diagnoses etc.

Extending to population projections

Given the population projections and the high hospitalisation rates with ID diagnoses in persons ≥ 80 years, we wanted to quantify the expected effect of projected ageing on the number of hospitalisations with ID diagnoses.

1. Age-specified hospitalisation rates of 2015-2019 were applied to projected populations in the same age strata. For example, in 2015-2019, the age-specific hospitalisation rate for ID diagnoses for the group aged 80+ years was 323 826 / 2 579 653 = 0.12553. According to projections, the number of person-years in this age group will be 5 126 333 in 2045 – 2049. This would entail 0.12553 * 5 126 333 = 643 509 hospitalisations with ID diagnoses in this age group alone.

2. These numbers were then added across age strata to obtain a total predicted number. This number was 46% higher than the number of 2015-2019. At the same time, the total population is projected to increase by 17%. The ageing of population will likely bring a disproportionate increase in hospitalisations with ID diagnoses.

3. This type of analysis relies on the assumptions that rates will remain the same and that population projections are correct. This is highly unlikely, and the analysis is meant as a crude way to visualise the potential impact of the ageing of the population on ID hospitalisation rates.

Sensitivity analyses

We performed sensitivity analyses since our classification is subjective and we wished to test its robustness. We also wanted to compare our classification to the classification from New Zealand by Baker et al. The following analyses were performed:

1. With the wider definition of intraabdominal infections, as discussed above.
2. With diagnoses removed from the original Baker classification as stated above (i.e., cancers, inflammatory disorders), but without any addition of diagnoses (i.e., Febrile neutropenia, pyrexia of unknown origin, etc. were not added).
3. Using all ICD-10-SE chapters, including mental disorders as well as maternal diagnoses, including normal delivery etc. In this analysis, IDs within Chapter XV – "Pregnancy, childbirth and the puerperium" were included as stated above, e.g., O85 – Puerperal sepsis.
4. The previous Baker classification, including cervical cancer, gastritis, Guillain-Barré etc.
5. Excluding transfers, including only hospitalisations where the patient was admitted from their homes. The rationale for this was to detect if changes in transfer patterns (e.g., an increased tendency to transfer post-operative infections from surgical wards) had influenced the results.
6. Excluding in-hospital mortality, including only patients discharged alive. The rationale was to detect changes in disease coding related to terminal disease (e.g., cancer patients dying from aspiration pneumonia).
7. Including unplanned admissions only. An unplanned admission is defined in the NPR as an admission for which no appointment has been made. The rationale was to restrict the analysis to acute healthcare events.

Results

The full classification of ICD-10 diagnoses is shown in table S3 and S4. The most frequent IDs are displayed in table S5. The most frequent diagnoses of each ID category are found in table S6. Hospitalisations included in the main analysis and excluded hospitalisations are displayed in table S7.

ID vs non-ID diagnoses

Annual hospitalisations and hospital nights are shown in table S8. Rates for each year for IDs and non-IDs are displayed in fig S6 (hospitalisations) and S7 (hospital nights).

Stratification by age and sex

Results stratified by age and sex are shown in table S9 for IDs and S10 for non-IDs. The hospitalisation rates for ID diagnoses by age-strata is shown in the manuscript.

Major diagnostic categories

Results for major ID categories are found in table S11 and in table S12 for non-ID categories. Figure S8 shows hospitalisation rate for diagnostic categories by age strata. Figure S9 shows SRR for hospital nights for ID vs major non-ID categories.

Rate of hospital nights per hospital bed and year

Yearly hospitalisations with ID and non-ID diagnoses divided by national bed capacity are shown in figure S10. (Equivalent to figure 5A in manuscript but on an annual basis).

Sensitivity analyses

1. When the wider definition of intraabdominal infections was used, the hospitalization rate with an ID diagnosis was higher, see table S13. The trend was slightly less pronounced. This indicates that intraabdominal infections increased slower than for the ID diagnoses in the main analysis.

2. When only removals (of secondary effects) were made from the Baker classification, the hospitalisation rate was lower and the trend less pronounced. This indicates that the increase for the diagnoses that were added to the previous classification was slightly above average. As seen above, the most prevalent diagnoses in this group were COPD with acute exacerbation, fever of unknown origin, aspiration pneumonia and febrile neutropenia.

3. When all ICD-10 chapters were included, the hospitalisation rate for non-ID diagnoses increased, as expected. Consequently, the proportion of hospital nights with an ID diagnosis decreased. As seen in table S12, hospitalisation rates increased for diagnosis within mental disorders and obstetrics, but with shorter LOS.

4. When the Baker classification was employed, the hospitalisation rate for IDs was similar to the main analysis in 2015-2019. However, the trend was less pronounced. This implies that the diagnoses that were removed from the original classification (i.e., secondary effects) did not have an increasing trend.

5. When transfers were removed from the analysis, the proportion of hospitalisations with ID diagnoses remained the same. However, the trend was slightly lower compared to the main analysis, possibly indicating an increased tendency to transfer patients with ID diagnoses.

6. When patients with in-hospital mortality were excluded, both hospitalisation rates and trends remained the same as in the main analysis. This indicates that there was no increased tendency to register ID diagnoses in terminal disease.

7. When only non-planned admissions were included in the analysis, the proportion of hospitalisations with ID diagnoses was higher. This was expected as ID hospitalisations would typically be unplanned. The trend was also more pronounced, indicating that ID hospitalisations were increasingly non-planned.

Figures

Figure S1. Population projection for Sweden

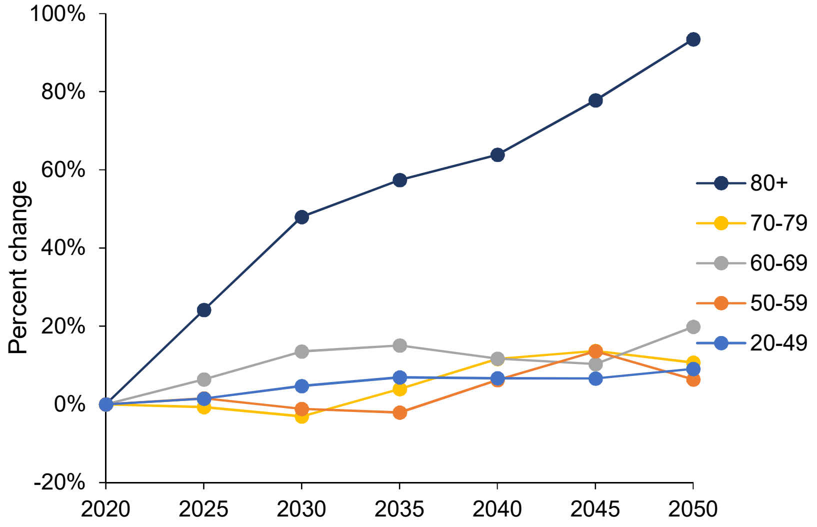

Figure S1. Projected percent change in numbers from 2020 for different age strata in the Swedish population. Data source: *Statistics Sweden*.

Figure S2. Population projection for the European Union

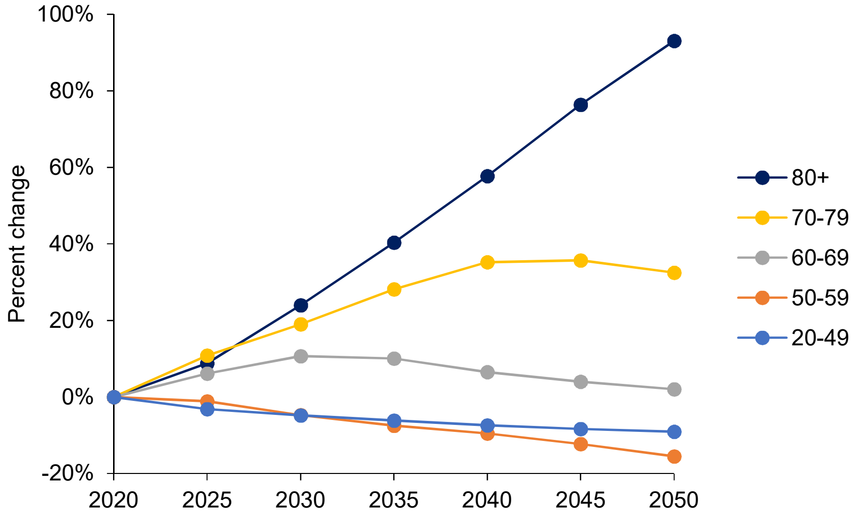

Figure S2. Projected percent change in numbers from 2020 for different age strata in population of the European Union. Data source: *Eurostat*

Figure S3. Population projection for the USA

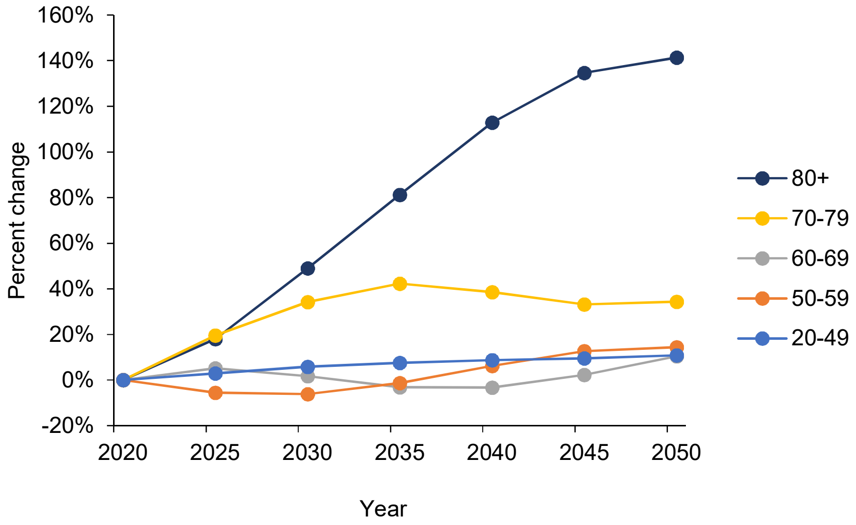

Figure S3. Projected percent change in numbers from 2020 for different age strata in the US population. Data source: *US Census Bureau*

Figure S4. Trends in hospital bed capacity in Sweden 1998 to 2019

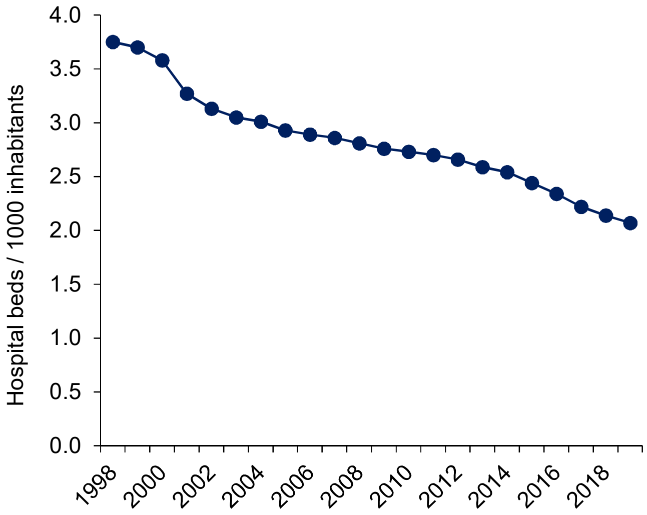

Figure S4. Hospital beds per 1000 inhabitants in Sweden from 1998 to 2018. Data source: *OECD Health statistics*

Figure S5. Trends in hospital bed capacity vs hospital use in Sweden 1998 to 2019

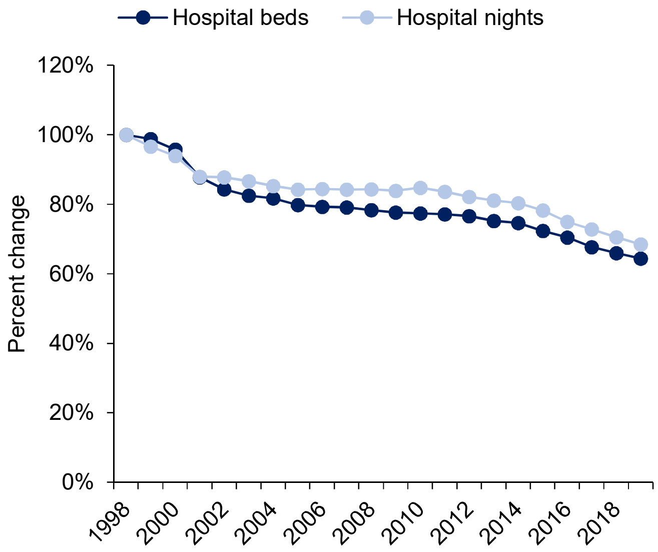

Figure S5. Trends, measured as percent change, from 1998 for hospital nights and hospital beds. *Data sources: OECD Health statistics (beds) and National Patient Register (hospital nights)*

Figure S6. Yearly hospitalisation rates for IDs and non-ID diagnoses

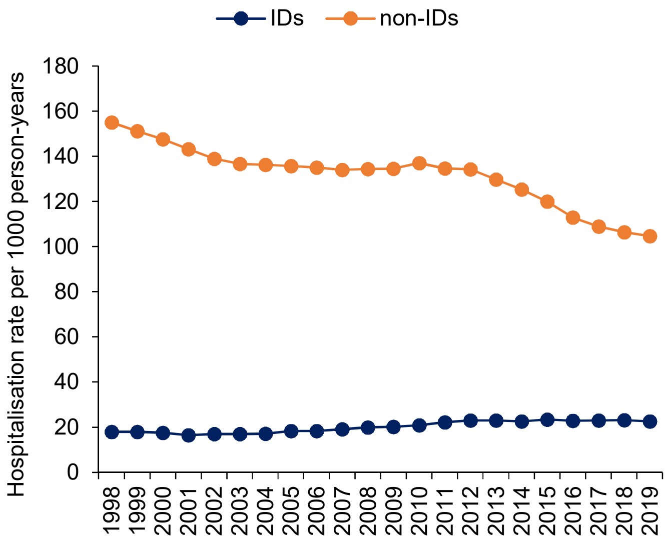

Figure S6. Yearly age-standardised hospitalisation rates for ID and non-ID diagnoses

Figure S7. Yearly rates for hospital nights for IDs and non-ID diagnoses

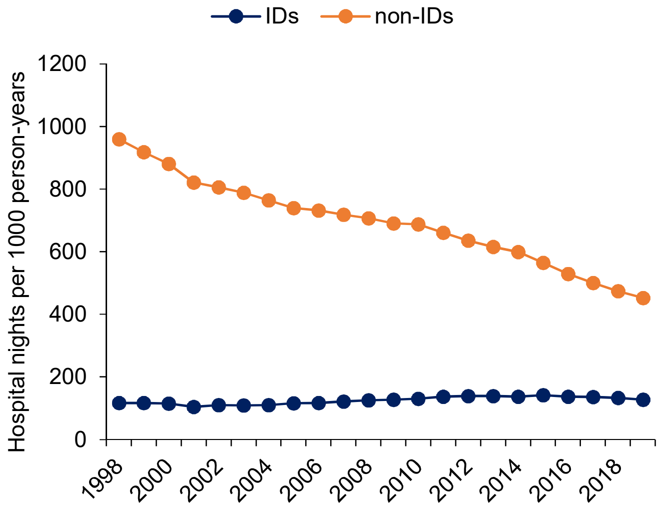

Figure S7. Yearly age-standardised rates for hospital nights for ID and non-ID diagnoses

Figure S8. Number of hospitalisations by major ID category and age 1998 to 2019

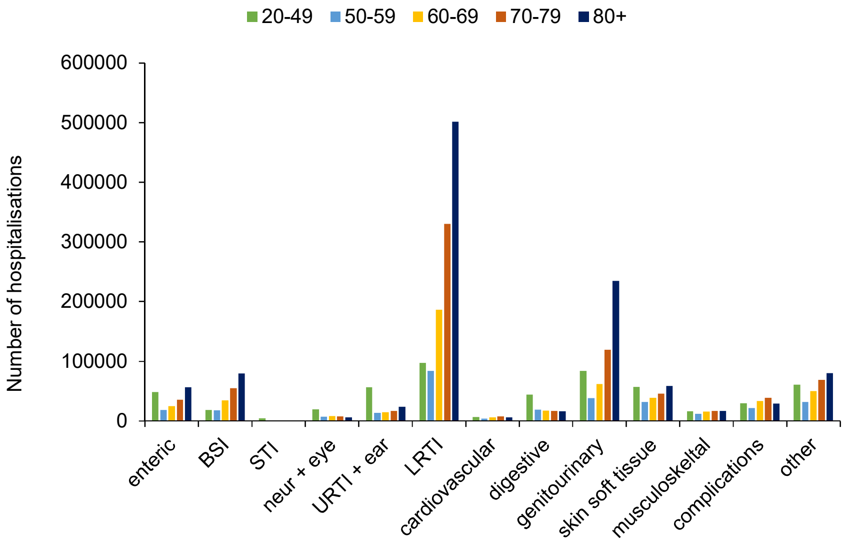

Figure S8. Number of hospitalisations for ID diagnosis groups 1998 – 2019, stratified by age. BSI = blood-stream infections, STI = sexually transmitted infections, URTI = upper respiratory tract infections, LRTI = lower respiratory tract infections

Figure S9. Standardised rate ratio for hospital nights for IDs vs major non-ID categories

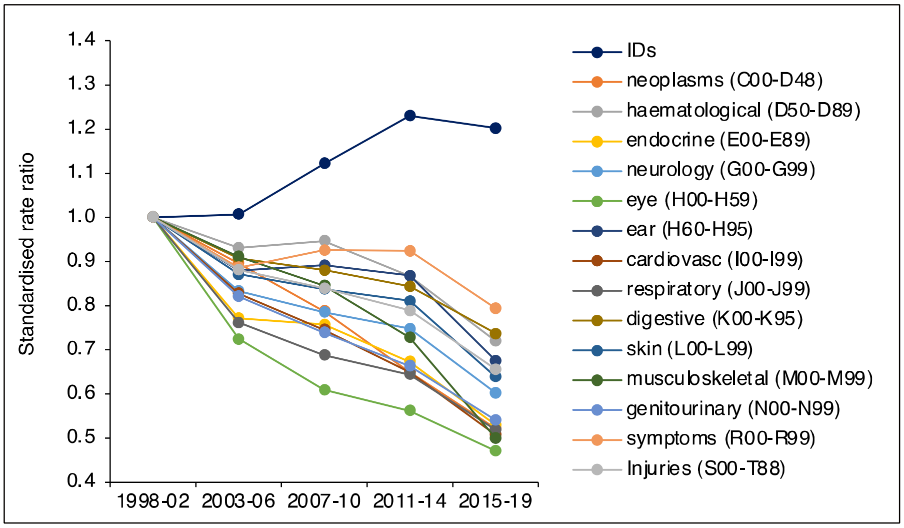

Figure S9. The number of hospital nights for diagnoses classified as IDs but found in other ICD-10 chapters have been subtracted from those chapters (e.g., the number of hospital nights with a pneumonia diagnosis, considered an ID, have been subtracted from respiratory (J00-J99)). Standardised rate ratio = ratio of age-standardised rate of hospital nights per 1000 person-years for each period vs the first period (1998 – 2002).

Figure S10. Rate ratio for the rate of hospital nights per bed and year for IDs and non-IDs

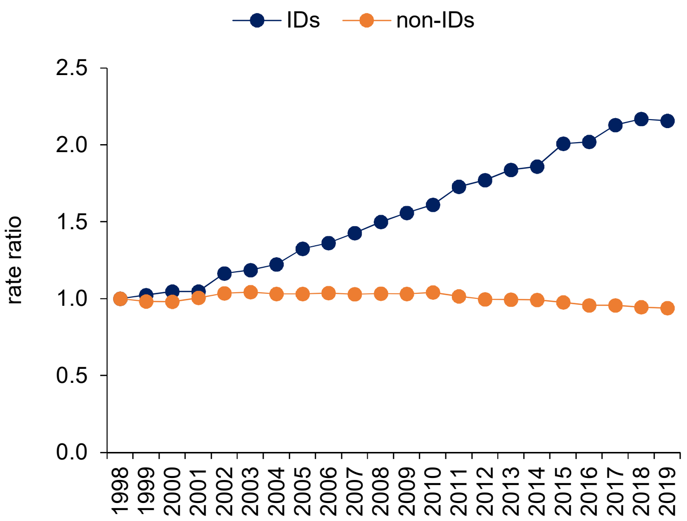

Figure S10. Trend for hospital nights for ID and non-ID diagnoses, adjusted by national bed capacity. (Equivalent to figure 5A in main manuscript, but on an annual basis)

Tables

Table S1. Studies of hospitalisation rates with ID diagnoses

First author	journal	publication year	country	study period	ID diagnosis
Fry	*JAMA*	2005	USA	1988 - 2002	Pneumonia
Thomsen	*JIM*	2006	Denmark	1994 - 2004	Pneumonia
Sögaard	*Resp Med*	2014	Denmark	1997 - 2011	Pneumonia
Quan	*Thorax*	2015	UK	1998 - 2014	Pneumonia
Simmering	*OFID*	2017	USA	1998 - 2011	Urinary tract
Akiyama	*BMJ Open*	2013	Japan	2007 - 2010	Osteomyelitis
Edelsberg	*Emerg Inf Dis*	2009	USA	2000 - 2004	Skin / soft tissue
Williamson	*BMC Inf Dis*	2013	N.Z.	2001 - 2011	Staph aureus
Kaye	*PLoS ONE*	2015	USA	2005 - 2011	Skin infections
Peterson	*OFID*	2017	USA	1998 - 2013	Cellulitis
Lopman	*CID*	2011	USA	1996 - 2007	Gastroenteritis
Pant	*JACC*	2015	USA	2000 - 2011	Endocarditis
Dayer	*Lancet*	2015	UK	2000 - 2013	Endocarditis
Erichsen	*Eur J Intern Med*	2016	Denmark	1994 - 2011	Endocarditis
Simonsen	*Arch Intern Med*	1998	USA	1980 - 1994	All IDs
Christensen	*CID*	2009	USA	1998 - 2006	All IDs
Baker	*Lancet*	2012	N.Z.	1989 - 2008	All IDs

Table S1. Studies of hospitalisation rates for different IDs by journal, publication year, country, study period and ID diagnosis. The last three studies are aggregating IDs from ICD codes.

Table S2. Coding issues for common ID diagnoses

code	diagnosis	issue	comment
J18.9	Pneumonia, unspecified	no issues	
N39.0	Urinary tract infection, site not specified	increase 2011	Change in sepsis coding guidelines
J15.9	Bacterial pneumonia, unspecified	increase 2005	Change in national guidelines
J44.1	COPD with acute exacerbation	increase 2007	Reciprocal decrease J440 and J209
N10.9	acute tubulo-interstitial nephritis	increase 2011	Change in sepsis coding guidelines
A46.9	Erysipelas	no issues	
T81.4	Infection following a procedure, not elsewhere classified	no issues	
A41.9	sepsis, unspecified	decrease in 2011	Change in sepsis coding guidelines
A09.9	gastroenteritis and colitis of unspecified origin	no issues	
J44.0	COPD with acute lower respiratory infection	decrease 2007	Reciprocal increase in J44.1
R50.9	Fever of unknown origin	no issue	
J20.9	Acute bronchitis, unspecified	large decrease	Reciprocal increase in J44.1
A41.5	Sepsis due to other gramnegative organisms	decrease in 2011	Change in sepsis coding guidelines
B99.9	Other and unspecified infectious diseases	no issue	
J06.9	Acute upper respiratory infection	large fluctuations	Annual flucuations in viral infections
B34.9	Viral infection, unspecified	no issue	
A04.7	Enterocolitis due to C.*Difficile*	no issue	
K61.0	Anal abscess	no issue	
J18.0	Bronchopneumonia, unspecified	large decrease	Change in national guidelines

Table S2. Potential coding issues found in graphical screening for ID diagnoses that individually contributed >1% to all ID hospitalisations, ranked (top-down) by overall hospitalisation rate 1998-2019. COPD = chronic obstructive pulmonary disorder

Table S3. ID diagnoses, by major ID category

Major ID category	Codes
Enteric infections	A00 – A09
Bloodstream infections	A40 – A41, R572, R650 – R651
Sexually transmitted infections	A50 – A64, B20 – B24
Infections of the neurological system, including the eye	A39, A80-A89, B30, G00 – G02, G039, G040 – G042, G049, G05-G08, G940, H000, H03, H043, H050, H061, H100, H102, H103, H109, H130, H131, H160, H162, H168, H169, H190 – H192, H220, H320, H440, H451
Upper respiratory tract infections, including the ear	H600-H603, H608, H609, H610, H62, H66, H67, H70, H730, H750, H830, H940, J00 – J06, J340, J36, J390, J391
Lower respiratory tract infections, including influenza	A15 – A19, A481, J09 – J22, J440 – J441, J690, J85 – J86
Infections of heart and blood vessels	I301, I320, I321, I33, I38, I39, I400, I410 – I412, I430, I520, I521, I681, I790, I791, I980, I981
Infections of the digestive system including the liver, narrow definition	B15 – B19, K044, K046, K047, K102, K113, K122, K230, K231, K61, K630, K650, K67, K750, K770, K830, K930, K931
Infections of the genitourinary system	N080, N10, N136, N151, N159, N160, N290, N291, N300, N309, N330, N340, N341, N37, N390, N410, N412 – N413, N431, N450, N459, N481, N482, N49, N51, N61, N70 – N74, N751, N764, N770, N771
Infections of skin and soft tissue	A46, L00 – L04, L050, L08, L303
Infections of bone, joints and connective tissue	M00 – M01, M462 – M465, M490 – M492, M600, M630 - M632, M650, M651, M680, M710, M711, M726, M730, M731, M86, M900 – M902
Infectious complications	T793, T802, T814, T826*, T827*, T835*, T836*, T845 – T847*, T857*, T874, T880
Other infections	A20 – A28, A30 – A38, A42 – A49, A65 – A69, A70 – A74, A75 – A79, A92 – A99, B00 – B09, B25 – B27, B33 – B99, R508, R509, D709, D733, E060, E321
**Included in sensitivity analysis only**	
Pregnancy, childbirth and the puerperium	O030, O035, O040, O045, O050, O055, O060, O065, O070, O075, O080, O23, O411, O753, O85, O86, O91, O98
Diseases of the digestive system – wide definition	above + K251, K252, K255, K256, K261, K262, K265, K266, K271, K272, K275, K276, K281, K282, K285, K286, K35, K401, K404, K411, K414, K421, K431, K434, K437, K441, K451, K461, K550, K56, K57, K800 – K804, K810, K819

Table S3. All Infectious disease diagnoses, by major category. The last two categories were included in sensitivity analysis 3 and 1, respectively. * = included in subcategory of device-associated infections.

Table S4. Diagnoses classified as non-IDs, by ICD-10 chapter

Chapter	Name of Chapter	Codes
II	Neoplasms	C00 – D48
III	Diseases of the blood and blood-forming organs and certain disorders involving the immune mechanism	D50 – D89, except those above
IV	Endocrine, nutritional and metabolic diseases	E00 – E90, except those above
VI	Diseases of the nervous system	G00 – G99, except those above
VII	Diseases of the eye and adnexa	H00 – H59, except those above
VIII	Diseases of the ear and mastoid process	H65 – H95, except those above
IX	Diseases of the circulatory system	I00 – I99, except those above
X	Diseases of the respiratory system	J00 – J99, except those above
XI	Diseases of the digestive system – narrow definition	K00 – K93, except those above
XII	Diseases of the skin and subcutaneous tissue	L00 – L99, except those above
XIII	Diseases of the musculoskeletal system and connective tissue	M00 – M99, except those above
XIV	Diseases of the genitourinary system	N00 – N99, except those above
XVIII	Symptoms, signs and abnormal clinical and laboratory findings, not elsewhere classified	R00 – R99, except those above
XIX	Injury, poisoning and certain other consequences of external causes	S00 – T98, except those above
	**Included in robustness analysis only**	
V	Mental and Behavioural disorders	F00 – F99
XV	Pregnancy, childbirth and the puerperium	O00 – O99, except those above
XVI	Certain conditions originating in the perinatal period	P00 – P96
XVII	Congenital malformations, deformations and chromosomal abnormalities	Q00 – Q99

Table S4. Non-infectious diagnosis, by ICD-10 chapter. The last four chapters were included in robustness analysis 3 only.

Table S5. The most frequent four-position ID diagnoses overall

code	diagnosis	rate 1998-02	% of IDs 1998-02	rate 2015-19	% of IDs 2015-19
J18.9	Pneumonia, unspecified	238	14.2%	255	10.9%
N39.0	Urinary tract infection, site not specified	124	7.2%	228	9.7%
J15.9	Bacterial pneumonia, unspecified	75	4.5%	201	8.6%
J44.1	COPD with acute exacerbation	84	5.3%	179	7.4%
N10.9	acute tubulo-interstitial nephritis	67	3.7%	125	5.7%
A46.9	Erysipelas	75	4.4%	95	4.0%
T81.4	Infection following a procedure, not elsewhere classified	36	2.0%	69	3.0%
A41.9	sepsis, unspecified	40	1.7%	55	2.4%
A09.9	gastroenteritis and colitis of unspecified origin	51	3.0%	39	1.6%
J44.0	COPD with acute lower respiratory infection	63	3.0%	13	0.6%
R50.9	Fever of unknown origin	35	2.5%	52	2.4%
J20.9	Acute bronchitis, unspecified	42	3.3%	20	0.6%
A41.5	Sepsis due to other gramnegative organisms	33	1.3%	23	1.0%
B99.9	Other and unspecified infectious diseases	30	1.3%	36	1.6%
J06.9	Acute upper respiratory infection	22	1.5%	32	1.2%
B34.9	Viral infection, unspecified	24	1.7%	29	1.2%
A04.7	Enterocolitis due to *C.Difficile*	19	1.0%	25	1.0%
K61.0	Anal abscess	18	0.9%	28	1.1%
J18.0	Bronchopneumonia, unspecified	33	3.2%	6	0.2%

Table S5. ID diagnoses that contributed by more than 1% of all ID hospitalisations, ranked by rate, over the entire period (1998 – 2019). Rates are hospitalisations per 100.000 person-years and % is the proportion of all ID hospitalisations of the first and last period, respectively. COPD = chronic obstructive pulmonary disorder.

Table S6. The most frequent codes in each ID major category

	ICD-10 code	diagnosis	% of category
**Enteric**	A09.9	Infectious gastroenteritis and colitis, unspecified	40%
	A04.7	Enterocolitis due to *Clostridium difficile*	20%
	A08.4	Viral intestinal infection, unspecified	8%
**BSI**	A41.9	Sepsis, unspecified organism	38%
	A41.5	Sepsis due to other Gram-negative organisms	25%
	A41.0	Sepsis due to *Staphylococcus aureus*	14%
**STI**	B24.9	Unspecified human immunodeficiency virus [HIV] disease	16%
	A60.0	Herpesviral infection of genitalia and urogenital tract	15%
	B23.8	HIV disease resulting in other specified conditions	11%
**Neuro + eye**	A87.9	Viral meningitis, unspecified	20%
	H16.9	Keratitis, unspecified	8%
	G06.0	Intracranial abscess and granuloma	8%
**URTI + ear**	J06.9	Acute upper respiratory infection, unspecified	37%
	J36.9	Peritonsillar abscess	12%
	J03.9	Acute tonsillitis, unspecified	11%
**LRTI + influenza**	J18.9	Pneumonia, unspecified	34%
	J15.9	Bacterial pneumonia	18%
	J44.1	COPD with acute exacerbation	17%
**Cardiovascular**	I33.0	Acute and subacute infective endocarditis	73%
	I33.9	Acute endocarditis, unspecified	9%
	I38.9	Endocarditis, valve unspecified	8%
**Digestive**	K61.0	Anal abscess	31%
	K83.0	Cholangitis	19%
	K65.0	Acute peritonitis	15%
**Genitourinary**	N39.0	Urinary tract infection, site not specified	53%
	N10.9	Acute tubulo-interstitial nephritis	27%
	N30.9	Cystitis, unspecified	6%
**Skin / soft tissue**	A46.9	Erysipelas	57%
	L02.4	Cutaneous abscess, furuncle and carbuncle of limb	9%
	L08.9	Local infection of skin and subcutaneous tissue, unspecified	7%
**Musculoskeletal**	M00.9	Pyogenic arthritis, unspecified	24%
	M00.0	Staphylococcal arthritis and polyarthritis	15%
	M86.9	Osteomyelitis, unspecified	10%
**Complications**	T81.4	Infection following a procedure, not elsewhere classified	57%
	T84.5	Infection and inflammatory reaction due to internal joint prosthesis	17%
	T84.6	Infection and inflammatory reaction due to internal fixation device [any site]	7%
**Other**	R50.9	Fever, unspecified	22%
	B99.9	Other and unspecified infectious diseases	18%
	B34.9	Viral infection, unspecified	12%

Table S6. The top three diagnosis codes in each major ID category, by overall hospitalisation rate (1998 – 2019). % of category = percent of hospitalisations within that ID category 1998 – 2019. BSI = blood-stream infections, STI = sexually transmitted infections, URTI = upper respiratory tract infections, LRTI = lower respiratory tract infections. COPD = chronic obstructive pulmonary disorder.

Table S7. Hospitalisations included and excluded from analysis

	1998-2002	2003-06	2007-10	2011-14	2015-19
**All adult hospitalisations**	1 330 782	1 310 388	1 379 450	1 438 342	1 348 668
**Excluded**					
Diagnosis missing	15 790	14 123	13 932	11 982	11 059
Mental and behavioural disorders	85 481	83 774	93 131	107 301	103 502
Maternal and perinatal conditions	112 563	120 699	129 901	132 840	136 748
Z diagnoses	41633	53 023	50 567	52 797	45 692
Codes for special purposes	3		29	66	137
**Included in analysis**	1 075 311	1 038 769	1 091 891	1 133 355	1 051 529
percentage of all hospitalisations	81%	79%	79%	79%	78%

Table S7. The average annual number of hospitalisations excluded and included for each period, respectively. Diagnosis missing = principal diagnosis not registered in the National Patient Register. Mental and behavioural disorders = ICD chapter V, Maternal and perinatal disorders = ICD chapter XV-XVII, Z diagnoses = ICD chapter XXII

Table S8. Hospitalisations and hospital nights, by year

year	ID hosp.	ID nights	non-ID hosp.	non-ID nights
1998	116 526	753 281	1 000 119	6 159 700
1999	117 123	758 163	979 577	5 922 379
2000	115 571	751 796	963 678	5 739 111
2001	109 959	688 697	940 809	5 388 201
2002	114 145	735 851	919 050	5 328 995
2003	114 258	733 582	910 284	5 253 881
2004	116 001	748 658	915 691	5 142 455
2005	125 681	793 575	920 652	5 025 523
2006	126 654	809 970	925 856	5 026 037
2007	133 694	846 487	927 618	4 975 171
2008	140 179	880 478	939 730	4 946 618
2009	143 814	906 266	952 477	4 891 806
2010	149 640	935 026	980 413	4 920 284
2011	160 929	998 661	973 594	4 781 091
2012	169 003	1 017 071	982 455	4 658 443
2013	171 498	1 036 469	961 520	4 567 022
2014	171 200	1 039 066	943 222	4 515 529
2015	179 016	1 089 514	915 422	4 317 235
2016	178 347	1 066 871	875 828	4 116 726
2017	182 004	1 080 375	857 520	3 952 958
2018	186 188	1 072 064	849 446	3 805 650
2019	184 717	1 041 480	849 155	3 689 892

Table S8. Hospitalisations and hospital nights for ID diagnoses (left) and non-ID diagnoses (right). hosp. = annual number of hospitalisations, nights = hospital nights.

Table S9. Hospitalisations with ID diagnoses, by age and sex

	measure	1998 – 2002	2003-06	2007-10	2011-14	2015-19	SRR (95%CI)
**20-49**	hosp	23	22	25	28	26	
	rate	6.6	6.1	7.0	7.3	6.6	**1.00 (1.00-1.00)**
	%	11%	12%	13%	13%	15%	
	nights	90	84	95	105	101	
	rate	25.3	23.6	26.6	27.6	25.6	**1.01 (1.01-1.01)**
	%	12%	13%	15%	17%	19%	
	LOS	3.8	3.9	3.8	3.8	3.9	
**50-59**	hosp	12	12	14	15	16	
	rate	9.8	10.0	11.3	12.7	12.4	**1.27 (1.26-1.28)**
	%	8%	9%	10%	12%	14%	
	nights	66	68	74	78	81	
	rate	54	56	61	66	64	**1.19 (1.18-1.19)**
	%	9%	11%	13%	15%	18%	
	LOS	5.5	5.6	5.4	5.2	5.2	
**60-69**	hosp	15	18	24	28	28	
	rate	18	19	23	24	24	**1.35 (1.34-1.36)**
	%	9%	10%	11%	13%	15%	
	nights	97	114	148	168	159	
	rate	117	118	146	144	141	**1.21 (1.21-1.21)**
	%	10%	11%	14%	16%	19%	
	LOS	6.5	6.3	6.2	5.9	5.8	
**70-79**	hosp	28	27	30	38	48	
	rate	39	40	46	50	52	**1.32 (1.31-1.32)**
	%	10%	11%	13%	15%	17%	
	nights	199	188	210	248	296	
	rate	283	284	318	327	319	**1.13 (1.13-1.13)**
	%	11%	13%	15%	18%	21%	
	LOS	7.2	7.0	6.9	6.5	6.2	
**80+**	hosp	37	42	49	59	65	
	rate	82	87	101	118	126	**1.54 (1.53-1.54)**
	%	13%	15%	16%	18%	21%	
	nights	286	318	366	423	432	
	rate	635	657	750	849	838	**1.32 (1.32-1.32)**
	%	13%	15%	17%	20%	24%	
	LOS	7.8	7.5	7.4	7.2	6.7	
**females**	hosp	58	60	70	83	87	
	rate	16.7	17.0	19.4	22.1	22.1	**1.32 (1.32-1.32)**
	%	10%	11%	13%	14%	17%	
	nights	373	388	443	503	507	
	rate	108	110	122	134	129	**1.19 (1.19-1.19)**
	%	11%	12%	15%	17%	20%	
	LOS	6.5	6.5	6.3	6.1	5.8	
**males**	hosp	57	61	71	86	95	
	rate	17.3	17.5	19.7	22.1	22.5	**1.30 (1.29-1.30)**
	%	10%	11%	13%	14%	17%	
	nights	364	383	449	520	563	
	rate	110	111	123	134	132	**1.19 (1.19-1.19)**
	%	12%	14%	16%	19%	22%	
	LOS	6.4	6.3	6.3	6.1	5.9	

Table S9. Hospitalisations with ID diagnoses, stratified by age and sex. hosp = average annual number of hospitalisations in period (thousands), rate = age-standardised rate per 1000 person-years, % = percent of all hospitalisations / hospital nights within that age strata or sex, respectively, LOS = average length-of-stay. SRR = ratio of age-standardised rates of the last period (2015 – 2019) vs the first (1998 – 2002).

Table S10. Hospitalisations with non-ID diagnoses, by age and sex

	measure	1998 – 02	2003-06	2007-10	2011-14	2015-19	SRR
**20-49**	hosp	181	166	172	179	146	
	rate	51	47	48	47	37	**0.72 (0.72-0.73)**
	nights	645	564	547	523	427	
	rate	182	159	154	137	108	**0.60 (0.59-0.60)**
	LOS	3.6	3.4	3.2	2.9	2.9	
**50 – 59**	hosp	137	124	116	112	99	
	rate	112	102	96	95	78	**0.70 (0.69-0.70)**
	nights	636	553	484	433	370	
	rate	519	454	402	369	292	**0.56 (0.56-0.56)**
	LOS	4.6	4.4	4.2	3.9	3.7	
**60 – 69**	hosp	160	172	193	192	156	
	rate	194	177	191	163	138	**0.71 (0.71-0.72)**
	nights	898	894	940	871	679	
	rate	1 086	922	927	743	603	**0.56 (0.55-0.56)**
	LOS	5.6	5.2	4.9	4.5	4.4	
**70 – 79**	hosp	241	209	210	219	228	
	rate	344	316	318	289	246	**0.72 (0.71-0.72)**
	nights	1 643	1 302	1 203	1 143	1 111	
	rate	2 345	1 966	1 821	1 510	1 197	**0.51 (0.51-0.51)**
	LOS	6.8	6.2	5.7	5.2	4.9	
**80+**	hosp	241	247	259	264	241	
	rate	535	510	531	529	467	**0.87 (0.87-0.87)**
	nights	1886	1799	1760	1660	1390	
	rate	4181	3718	3608	3331	2694	**0.64 (0.64-0.64)**
	LOS	7.8	7.3	6.8	6.3	5.8	
**females**	hosp	500	474	491	495	439	
	rate	145	134	135	131	111	**0.77 (0.77-0.77)**
	nights	3 084	2 727	2 603	2 407	2 018	
	rate	890	766	712	639	509	**0.57 (0.57-0.57)**
	LOS	6.2	5.8	5.3	4.9	4.6	
**males**	hosp	460	444	459	470	431	
	rate	139	128	125	121	102	**0.73 (0.73-0.73)**
	nights	2 624	2 385	2 331	2 223	1 959	
	rate	789	682	630	565	456	**0.58 (0.58-0.58)**
	LOS	5.7	5.4	5.1	4.7	4.5	

Table S10. Hospitalisations with non-ID diagnoses, stratified by age and sex. hosp = average annual number of hospitalisations in period (thousands), rate = age-standardised rate per 1000 person-years, LOS = average length-of-stay. SRR = ratio of age-standardised rates of the last period (2015 – 2019) vs the first (1998 – 2002).

Table S11. Hospitalisations with ID diagnoses, by major category

iD category	measure	1998 – 2002	2003-06	2007-10	2011-14	2015-18	SRR
**enteric**	hosp	7	7	9	10	8	
	rate	1.1	1.1	1.3	1.3	1.1	**0.97 (0.95-0.98)**
	%	6.4%	6.1%	6.5%	5.9%	4.6%	
	nights	33	34	42	44	36	
	rate	4.9	4.9	5.9	6.0	4.6	**0.93 (0.92-0.93)**
	%	4.4%	4.4%	4.7%	4.3%	3.4%	
	LOS	4.5	4.6	4.6	4.4	4.4	
**BSI**	hosp	7	9	12	9	10	
	rate	1.0	1.4	1.7	1.3	1.2	**1.21 (1.19-1.22)**
	%	5.8%	7.7%	8.5%	5.6%	5.4%	
	nights	63	84	104	79	78	
	rate	10	12	15	11	10	**1.02 (1.01-1.02)**
	%	8.5%	10.9%	11.6%	7.7%	7.2%	
	LOS	9.4	9.0	8.6	8.3	7.9	
**STI**	hosp	0.4	0.3	0.3	0.3	0.2	
	rate	0.06	0.05	0.05	0.03	0.02	**0.40 (0.37-0.43)**
	%	0.3%	0.3%	0.2%	0.2%	0.1%	
	nights	3.0	2.6	3.0	2.6	1.6	
	rate	0.44	0.37	0.43	0.36	0.21	**0.49 (0.47-0.50)**
	%	0.4%	0.3%	0.3%	0.3%	0.1%	
	LOS	7.7	7.7	8.9	10.2	9.1	
**Neuro + eye**	hosp	2.0	2.0	2.1	2.5	2.5	
	rate	0.30	0.29	0.30	0.34	0.31	**1.06 (1.03-1.09)**
	%	1.7%	1.6%	1.5%	1.5%	1.3%	
	nights	17	17	18	21	21	
	rate	2.5	2.4	2.6	2.9	2.7	**1.08 (1.07-1.09)**
	%	2.2%	2.2%	2.0%	2.1%	2.0%	
	LOS	8.3	8.4	8.5	8.5	8.6	
**URTI + ear**	hosp	5.6	5.0	5.8	6.3	5.8	
	rate	0.82	0.73	0.80	0.84	0.74	**0.90 (0.88-0.91)**
	%	4.9%	4.2%	4.1%	3.7%	3.2%	
	nights	17	15	17	19	18	
	rate	2.6	2.2	2.4	2.6	2.3	**0.87 (0.86-0.88)**
	%	2.4%	2.0%	2.0%	1.9%	1.7%	
	LOS	3.1	3.0	3.0	3.0	3.1	
**LRTI + influenza**	hosp	44	45	52	62	69	
	rate	6.8	6.6	7.3	8.4	8.6	**1.27 (1.26-1.27)**
	%	38.8%	36.9%	36.3%	37.1%	37.7%	
	nights	308	299	335	390	406	
	rate	47	44	47	53	51	**1.08 (1.08-1.08)**
	%	41.8%	38.8%	37.5%	38.1%	37.9%	
	LOS	6.9	6.7	6.5	6.2	5.9	
**Cardiovascular**	hosp	0.9	1.0	1.3	1.7	1.9	
	rate	0.13	0.16	0.18	0.22	0.25	**1.83 (1.76-1.90)**
	%	0.8%	0.9%	0.9%	1.0%	1.1%	
	nights	14	16	19	24	29	
	rate	2.1	2.4	2.7	3.3	3.7	**1.79 (1.77-1.80)**
	%	1.8%	2.1%	2.1%	2.4%	2.7%	
	LOS	15.3	15.2	14.7	14.8	15.1	
**Digestive**	hosp	4.2	4.4	5.1	5.8	6.3	
	rate	0.63	0.65	0.72	0.79	0.81	**1.28 (1.26-1.31)**
	%	3.7%	3.7%	3.6%	3.5%	3.4%	
	nights	23	24	27	29	31	
	rate	3.4	3.6	3.8	3.9	3.9	**1.14 (1.13-1.15)**
	%	3.1%	3.1%	3.0%	2.8%	2.9%	
	LOS	5.4	5.5	5.2	5.0	4.9	
**Genitourinary**	hosp	18	20	22	29	33	
	rate	2.7	2.9	3.1	3.9	4.1	**1.55 (1.53-1.56)**
	%	15.6%	16.5%	15.4%	17.5%	18.1%	
	nights	94	104	113	151	163	
	rate	14	15	16	20	20	**1.45 (1.44-1.45)**
	%	12.8%	13.5%	12.6%	14.8%	15.2%	
	LOS	5.3	5.2	5.2	5.1	4.9	
**Skin /soft tissue**	hosp	8.7	8.7	10	12	13	
	rate	1.3	1.3	1.4	1.6	1.7	**1.28 (1.26-1.29)**
	%	7.6%	7.2%	7.0%	7.2%	7.2%	
	nights	49	48	54	66	69	

	rate	7.4	7.0	7.6	8.9	8.8	**1.18 (1.18-1.19)**
	%	6.6%	6.2%	6.1%	6.4%	6.5%	
	LOS	5.6	5.5	5.4	5.4	5.3	
**Musculoskeletal**	hosp	2.9	3.0	3.6	3.9	4.1	
	rate	0.44	0.44	0.51	0.53	0.53	**1.21 (1.18-1.23)**
	%	2.5%	2.5%	2.5%	2.3%	2.3%	
	nights	25	26	33	37	40	
	rate	3.8	3.9	4.7	5.1	5.0	**1.32 (1.31-1.33)**
	%	3.4%	3.4%	3.7%	3.7%	3.7%	
	LOS	8.7	8.8	9.2	9.6	9.6	
**Complications**	hosp	3.8	4.9	6.7	9.0	10.3	
	rate	0.58	0.74	0.96	1.2	1.3	**2.26 (2.22-2.30)**
	%	3.3%	4.1%	4.8%	5.3%	5.6%	
	nights	33	42	58	75	82	
	rate	5.2	6.4	8.4	10.2	10.5	**2.02 (2.01-2.03)**
	%	4.5%	5.5%	6.5%	7.3%	7.7%	
	LOS	8.8	8.6	8.6	8.3	8.0	
**Other**	hosp	10	10	12	15	18	
	rate	1.5	1.5	1.7	2.1	2.3	**1.52 (1.51-1.54)**
	%	8.7%	8.3%	8.6%	9.2%	10.0%	
	nights	59	59	69	85	96	
	rate	9.0	8.7	9.8	12	12	**1.34 (1.34-1.35)**
	%	8.0%	7.6%	7.8%	8.3%	9.0%	
	LOS	5.9	5.8	5.6	5.5	5.3	

Table S11. Hospitalisations with ID diagnoses, stratified by major category. hosp = average annual number of hospitalisations in period (thousands), rate = age-standardised rate per 1000 person-years, % = percent of all ID hospitalisations / hospital nights, respectively, LOS = average length-of-stay. SRR = ratio of age-standardised rates of the last period (2015 – 2019) vs the first (1998 – 2002). BSI = blood-stream infections, STI = sexually transmitted infections, URTI = upper respiratory tract infections, LRTI = lower respiratory tract infections.

Table S11B. Subcategorisation of device-associated infections

iD category	measure	1998 – 2002	2003-06	2007-10	2011-14	2015-18	SRR
**Subgroup**	hosp	1.4	2.0	2.6	3.7	4.5	
**Device-associated**	rate	0.21	0.29	0.38	0.50	0.57	**2.72 (2.65-2.79)**
	%	1.2%	1.6%	1.9%	2.2%	2.5%	
	nights	15	21	29	38	45	
	rate	2.4	3.2	4.1	5.2	5.7	**2.38 (2.36-2.39)**
	%	2.1%	2.7%	3.2%	3.7%	4.2%	
	LOS	11.4	10.8	10.9	10.4	10.0	

Table S11B. Subgroup of infectious complications, displaying diagnoses of device-associated infections. This group comprises of a composite of ICD-10 codes T82.6, T82.7, T83.5, T83.6, T84.5, T84.6, T84.7, T85.7. hosp = average annual number of hospitalisations in period (thousands), rate = age-standardised rate per 1000 person-years, % = percent of all ID hospitalisations / hospital nights, respectively, LOS = average length-of-stay. SRR = ratio of age-standardised rates of the last period (2015 – 2019) vs the first (1998 – 2002).

Table S12. Hospitalisations with non-ID diagnoses, by ICD-10-SE chapter

non-ID category	measure	1998 – 2002	2003-06	2007-10	2011-14	2015-18	SRR
**neoplasms**	hosp	141	131	125	117	105	
**C00 – D48**	rate	22	20	18	16	13	**0.61 (0.61-0.61)**
	nights	1082	1006	926	804	694	
	rate	169	152	133	110	88	**0.52 (0.52-0.52)**
	LOS	7.7	7.7	7.4	6.9	6.6	
**haematological**	hosp	9	10	12	12	11	
**D50 – D89**	rate	1.4	1.5	1.6	1.6	1.4	**0.98 (0.96-0.99)**
	nights	52	51	53	51	45	
	rate	7.9	7.3	7.5	6.8	5.7	**0.72 (0.72-0.72)**
	LOS	5.5	4.9	4.6	4.3	4.1	
**endocrine**	hosp	27	25	29	32	28	
**E00 – E90**	rate	4.2	3.7	4.0	4.4	3.6	**0.87 (0.86-0.88)**
	nights	188	151	154	144	120	
	rate	29	22	22	19	15	**0.53 (0.53-0.53)**
	LOS	6.9	6.1	5.4	4.5	4.3	
**neurologic**	hosp	37	34	39	41	36	
**G00 – G99**	rate	5.7	5.1	5.5	5.6	4.6	**0.81 (0.80-0.81)**
	nights	242	209	204	204	176	
	rate	37	31	29	28	22	**0.60 (0.60-0.60)**
	LOS	6.5	6.1	5.3	5.0	4.9	
**eye**	hosp	9.3	7.7	7.6	7.8	7.5	
**H00 – H59**	rate	1.4	1.2	1.1	1.1	1.0	**0.67 (0.67-0.68)**
	nights	27	20	18	17	15	
	rate	4.2	3.0	2.5	2.3	2.0	**0.47 (0.47-0.47)**
	LOS	2.9	2.6	2.3	2.2	2.0	
**ear**	hosp	5.6	5.8	6.3	6.6	5.8	
**H60 – H95**	rate	0.8	0.9	0.9	0.9	0.7	**0.88 (0.86-0.89)**
	nights	15	14	15	15	12	
	rate	2.3	2.1	2.1	2.0	1.6	**0.68 (0.67-0.68)**
	LOS	2.8	2.4	2.3	2.3	2.1	
**cardiovasc**	hosp	248	233	234	225	198	
**I00 – I99**	rate	38	35	33	31	25	**0.65 (0.65-0.65)**
	nights	1629	1408	1322	1206	1009	
	rate	251	208	187	163	127	**0.51 (0.51-0.51)**
	LOS	6.6	6.0	5.6	5.4	5.1	
**respiratory**	hosp	28	24	22	22	19	
**J00 – J99**	rate	4.3	3.5	3.2	3.0	2.4	**0.56 (0.55-0.56)**
	nights	163	129	122	120	105	
	rate	25	19	18	16	13	**0.52 (0.52-0.52)**
	LOS	5.8	5.5	5.4	5.5	5.5	
**digestive**	hosp	101	98	101	109	104	
**K00 – K93**	rate	15	14	14	15	13	**0.87 (0.86-0.87)**
	nights	500	469	473	473	440	
	rate	76	69	67	64	56	**0.74 (0.74-0.74)**
	LOS	4.9	4.8	4.7	4.3	4.2	
**skin**	hosp	5.8	5.6	5.7	6.4	5.7	
**L00 – L99**	rate	0.9	0.8	0.8	0.9	0.7	**0.83 (0.81-0.84)**
	nights	52	47	47	48	40	
	rate	8.0	6.9	6.7	6.4	5.1	**0.64 (0.63-0.64)**
	LOS	8.9	8.5	8.2	7.4	7.0	
**musculoskeletal**	hosp	69	71	81	86	79	
**M00 – M99**	rate	11	11	12	12	10	**0.95 (0.95-0.95)**
	nights	463	436	423	383	282	
	rate	72	65	61	52	36	**0.50 (0.50-0.50)**
	LOS	6.7	6.1	5.2	4.5	3.6	
**urogenital**	hosp	47	43	43	42	38	
**N00 – N99**	rate	7.1	6.4	6.1	5.7	4.9	**0.68 (0.68-0.68)**
	nights	202	171	161	151	132	
	rate	31	26	23	21	17	**0.54 (0.54-0.54)**
	LOS	4.3	4.0	3.7	3.6	3.5	
**symptoms**	hosp	118	116	126	134	116	
**R00 – R98**	rate	18	17	18	18	15	**0.82 (0.82-0.82)**
	nights	330	303	328	343	314	
	rate	50	44	46	46	40	**0.79 (0.79-0.79)**
	LOS	2.8	2.6	2.6	2.6	2.7	
**injuries**	hosp	114	114	119	125	117	
**S00 – T98**	rate	17	16	17	17	15	**0.87 (0.87-0.87)**
	nights	762	698	688	671	594	
	rate	114	100	96	90	75	**0.66 (0.65-0.66)**
	LOS	6.7	6.1	5.8	5.4	5.1	

**In sensitivity analysis only**	**measure**	**1998 – 2002**	**2003-06**	**2007-10**	**2011-14**	**2015-19**	**SRR**
**psychiatric**	hosp	85	84	93	107	104	
**F00 - F99**	rate	13	12	13	14	13	**1.05 (1.05-1.06)**
	nights	1619	1403	1536	1412	1525	
	rate	238	203	216	190	197	**0.82 (0.82-0.83)**
	LOS	19	17	17	13	15	
**obstetric**	hosp	108	117	126	128	132	
**O00 - O99**	rate	15	16	17	17	17	**1.11 (1.11-1.11)**
	nights	348	332	327	317	305	
	rate	48	46	45	42	38	**0.80 (0.80-0.80)**
	LOS	3.2	2.8	2.6	2.5	2.3	
**perinatal**	hosp	0.1	0.0	0.0	0.0	0.0	
**P00 - P99**	rate	0.02	0.01	0.00	0.00	0.00	**0.09 (0.07-0.11)**
	nights	1.1	0.8	0.2	0.1	0.1	
	rate	0.17	0.11	0.03	0.01	0.01	**0.05 (0.04-0.06)**
	LOS	8.8	17.2	6.5	5.8	5.5	
**congenital**	hosp	2.6	2.4	2.5	2.8	2.7	
**Q00 - Q99**	rate	0.37	0.35	0.35	0.37	0.35	**0.94 (0.92-0.96)**
	nights	13	11	11	11	9	
	rate	1.9	1.6	1.5	1.4	1.1	**0.59 (0.58-0.60)**
	LOS	5.0	4.7	4.3	3.8	3.2	
**Other**	hosp	42	53	51	53	46	
**Z00 - Z99**	rate	6.3	7.9	7.2	7.2	5.8	**0.92 (0.92-0.93)**
	nights	283	331	290	215	201	
	rate	43	49	41	29	26	**0.60 (0.60-0.60)**
	LOS	6.6	6.2	5.5	4.1	4.4	

Table S12. Hospitalisations with non-ID diagnoses, stratified by ICD-10-SE chapter. hosp = average annual number of hospitalisations in period (thousands), rate = age-standardised rate per 1000 person-years, LOS = average length-of-stay. SRR = ratio of age-standardised rates of the last period (2015 – 2019) vs the first (1998 – 2002).

Table S13 Sensitivity analyses

	measure	1998 – 2002	2003-06	2007-10	2011-14	2015-19	SRR
**ORIGINAL**	hosp	115	121	142	168	182	
**IDS**	rate	17.4	17.7	20.0	22.7	23.0	**1.32 (1.32-1.33)**
	%	11%	12%	13%	15%	17%	
	nights	738	771	892	1 023	1 070	
	rate	112	113	126	138	135	**1.20 (1.20-1.20)**
	%	11%	13%	15%	18%	21%	
	LOS	6.4	6.4	6.3	6.1	5.9	
**non-IDs**	hosp	961	918	950	965	869	
	rate	147	136	135	131	110	**0.75 (0.75-0.75)**
	nights	5708	5112	4933	4631	3976	
	rate	877	756	700	627	503	**0.57 (0.57-0.75)**
	LOS	5.9	5.6	5.2	4.8	4.6	
**analysis 1**	hosp	145	151	175	205	221	
**IDS**	rate	21.9	22.1	24.7	27.7	27.9	**1.28 (1.27-1.28)**
	%	13%	15%	16%	18%	21%	
	nights	898	926	1 052	1 190	1 234	
	rate	137	136	149	161	156	**1.14 (1.14-1.14)**
	%	14%	16%	18%	21%	24%	
**non-IDs**	LOS	6.4	6.4	6.3	6.1	5.9	
	hosp	931	888	917	928	831	
	rate	143	131	130	126	106	**0.74 (0.74-0.74)**
	nights	5548	4957	4774	4464	3812	
	rate	852	733	678	605	482	**0.57 (0.57-0.57)**
	LOS	6.0	5.6	5.2	4.8	4.6	
**analysis 2**	hosp	109	114	133	156	168	
**IDs**	rate	16.5	16.6	18.8	21.0	21.3	**1.29 (1.28-1.29)**
	%	10%	11%	12%	14%	16%	
	nights	700	729	840	949	993	
	rate	106	107	118	128	125	**1.18 (1.18-1.18)**
	%	11%	12%	14%	17%	20%	
	LOS	6.4	6.4	6.3	6.1	5.9	
**non-IDs**	hosp	966	925	959	978	883	
	rate	148	137	136	133	112	**0.76 (0.76-0.76)**
	nights	5745	5154	4985	4705	4053	
	rate	883	763	708	637	513	**0.58 (0.58-0.58)**
	LOS	5.9	5.6	5.2	4.8	4.6	
**analysis 3**	hosp	117	122	144	170	184	
**IDs**	rate	17.6	17.9	20.3	23.0	23.3	**1.32 (1.32-1.32)**
	%	9%	9%	11%	12%	14%	
	nights	743	776	897	1 028	1 076	
	rate	113	114	127	139	136	**1.20 (1.20-1.20)**
	%	9%	10%	11%	14%	15%	
	LOS	6.5	6.4	6.3	6.1	5.9	
**non-IDs**	hosp	1198	1174	1222	1256	1153	
	rate	181	172	173	170	146	**0.81 (0.81-0.81)**
	nights	7971	7189	7098	6585	6016	
	rate	1208	1056	1004	890	764	**0.63 (0.63-0.63)**
	LOS	6.7	6.1	5.8	5.2	5.2	
**analysis 4**	hosp	135	136	156	180	191	
**IDs**	rate	20.4	19.9	22.0	24.2	24.1	**1.18 (1.18-1.19)**
	%	13%	13%	14%	16%	18%	
	nights	827	836	942	1 044	1 077	
	rate	126	122	133	141	136	**1.08 (1.08-1.08)**
	%	13%	14%	16%	18%	21%	
	LOS	6.1	6.1	6.0	5.8	5.6	
**non-IDs**	hosp	940	903	936	954	861	
	rate	144	134	133	129	109	**0.76 (0.76-0.76)**
	nights	5618	5047	4883	4610	3970	
	rate	863	747	694	624	502	**0.58 (0.58-0.58)**
	LOS	6.0	5.6	5.2	4.8	4.6	
**analysis 5**	hosp	100	105	121	144	151	
**IDs**	rate	15.2	15.4	17.0	19.4	19.1	**1.26 (1.25-1.26)**
	%	10%	11%	13%	14%	17%	
	nights	591	619	691	799	811	
	rate	90	91	98	108	102	**1.14 (1.13-1.14)**
	%	12%	13%	15%	18%	21%	
	LOS	5.9	5.9	5.7	5.6	5.4	
**non-IDs**	hosp	860	812	830	851	752	
	rate	132	120	118	115	96	**0.73 (0.73-0.73)**
	nights	4506	4042	3799	3598	3006	
	rate	692	598	539	488	380	**0.55 (0.55-0.55)**

	LOS	5.2	5.0	4.6	4.2	4.0	
**analysis 6**	hosp	110	116	136	162	175	
**IDs**	rate	16.7	17.0	19.3	21.8	22.1	**1.32 (1.32-1.32)**
	%	11%	12%	13%	15%	17%	
	nights	696	729	844	967	1 015	
	rate	106	107	119	131	128	**1.21 (1.21-1.21)**
	%	12%	13%	15%	18%	21%	
	LOS	6.3	6.3	6.2	6.0	5.8	
**non-IDs**	hosp	929	889	922	938	844	
	rate	142	132	131	127	107	**0.75 (0.75-0.75)**
	nights	5350	4791	4646	4369	3738	
	rate	822	709	660	592	473	**0.58 (0.58-0.58)**
	LOS	5.8	5.4	5.0	4.7	4.4	
**analysis 7**	hosp	102	108	129	157	169	
**IDs**	rate	15.4	15.8	18.2	21.2	21.3	**1.38 (1.38-1.39)**
	%	14%	15%	16%	18%	21%	
	nights	625	659	778	925	963	
	rate	95	97	110	125	121	**1.28 (1.28-1.28)**
	%	15%	17%	19%	22%	25%	
	LOS	6.1	6.1	6.0	5.9	5.7	
**non-IDs**	hosp	631	609	661	695	625	
	rate	96	90	94	94	79	**0.82 (0.82-0.82)**
	nights	3594	3279	3354	3334	2907	
	rate	550	482	474	450	367	**0.67 (0.67-0.67)**
	LOS	5.7	5.4	5.1	4.8	4.7	

Table S13. Sensitivity analyses. hosp = average annual number of hospitalisations in period (thousands), rate = age-standardised rate per 1000 person-years, % = percent of all hospitalisations / hospital nights with an ID diagnosis, LOS = average length-of-stay. SRR = ratio of age-standardised rates of the last period (2015 – 2019) vs the first (1998 – 2002). Analysis 1 = with wider definition of intra-abdominal infections, analysis 2 = Using the Baker classification without additions, analysis 3 = including all chapters (also maternal and psychiatric disorders etc.), analysis 4 = using the classification by Baker et al., analysis 5 = excluding transfers, analysis 6 = including only patients surviving at discharge, analysis 7 = including only unplanned admissions.

References

1. Statistical Database - Population statistics [Internet]. Stockholm: Statistics Sweden; 1860-. [cited 2020 Feb 9]. Available from: http://www.statistikdatabasen.scb.se/pxweb/en/ssd/
2. Organisation for economic cooperation and development (OECD), Health at a Glance 2019: OECD Indicators, OECD Publishing, Paris, https://doi.org/10.1787/4dd50c09-en.
3. Projected 5-Year Age Groups and Sex Composition: Main Projections Series for the United States, 2017-2060. U.S. Census Bureau, Population Division: Washington, DC.
4. Dayer MJ, Jones S, Prendergast B, Baddour LM, Lockhart PB, Thornhill MH. Incidence of infective endocarditis in England, 2000-13: a secular trend, interrupted time-series analysis. *Lancet* 2015; **385**(9974): 1219-28.
5. Edelsberg J, Taneja C, Zervos M, et al. Trends in US hospital admissions for skin and soft tissue infections. *Emerg Infect Dis* 2009; **15**(9): 1516-8.
6. Fry AM, Shay DK, Holman RC, Curns AT, Anderson LJ. Trends in hospitalizations for pneumonia among persons aged 65 years or older in the United States, 1988-2002. *JAMA* 2005; **294**(21): 2712-9.
7. Kaye KS, Patel DA, Stephens JM, Khachatryan A, Patel A, Johnson K. Rising United States Hospital Admissions for Acute Bacterial Skin and Skin Structure Infections: Recent Trends and Economic Impact. *PLoS One* 2015; **10**(11): e0143276.
8. Lopman BA, Hall AJ, Curns AT, Parashar UD. Increasing rates of gastroenteritis hospital discharges in US adults and the contribution of norovirus, 1996-2007. *Clin Infect Dis* 2011; **52**(4): 466-74.
9. Naucler P, Henriques-Normark B, Hedlund J, Galanis I, Granath F, Ortqvist A. The changing epidemiology of community-acquired pneumonia: nationwide register-based study in Sweden. *J Intern Med* 2019; **286**(6): 689-701.
10. Pant S, Patel NJ, Deshmukh A, et al. Trends in infective endocarditis incidence, microbiology, and valve replacement in the United States from 2000 to 2011. *J Am Coll Cardiol* 2015; **65**(19): 2070-6.
11. Peterson RA, Polgreen LA, Cavanaugh JE, Polgreen PM. Increasing Incidence, Cost, and Seasonality in Patients Hospitalized for Cellulitis. *Open Forum Infect Dis* 2017; **4**(1): ofx008.
12. Quan TP, Fawcett NJ, Wrightson JM, et al. Increasing burden of community-acquired pneumonia leading to hospitalisation, 1998-2014. *Thorax* 2016; **71**(6): 535-42.
13. Reveles KR, Lee GC, Boyd NK, Frei CR. The rise in Clostridium difficile infection incidence among hospitalized adults in the United States: 2001-2010. *Am J Infect Control* 2014; **42**(10): 1028-32.
14. Simmering JE, Tang F, Cavanaugh JE, Polgreen LA, Polgreen PM. The Increase in Hospitalizations for Urinary Tract Infections and the Associated Costs in the United States, 1998-2011. *Open Forum Infect Dis* 2017; **4**(1): ofw281.
15. Søgaard M, Nielsen RB, Schønheyder HC, Nørgaard M, Thomsen RW. Nationwide trends in pneumonia hospitalization rates and mortality, Denmark 1997-2011. *Respir Med* 2014; **108**(8): 1214-22.
16. Thomsen RW, Riis A, Nørgaard M, et al. Rising incidence and persistently high mortality of hospitalized pneumonia: a 10-year population-based study in Denmark. *J Intern Med* 2006; **259**(4): 410-7.
17. van Gageldonk-Lafeber AB, Bogaerts MA, Verheij RA, van der Sande MA. Time trends in primary-care morbidity, hospitalization and mortality due to pneumonia. *Epidemiol Infect* 2009; **137**(10): 1472-8.
18. Vihta KD, Stoesser N, Llewelyn MJ, et al. Trends over time in Escherichia coli bloodstream infections, urinary tract infections, and antibiotic susceptibilities in Oxfordshire, UK, 1998-2016: a study of electronic health records. *Lancet Infect Dis* 2018; **18**(10): 1138-49.
19. Baker MG, Barnard LT, Kvalsvig A, et al. Increasing incidence of serious infectious diseases and inequalities in New Zealand: a national epidemiological study. *Lancet* 2012; **379**(9821): 1112-9.
20. Christensen KL, Holman RC, Steiner CA, Sejvar JJ, Stoll BJ, Schonberger LB. Infectious disease hospitalizations in the United States. *Clin Infect Dis* 2009; **49**(7): 1025-35.
21. Pinner RW, Teutsch SM, Simonsen L, et al. Trends in infectious diseases mortality in the United States. *JAMA* 1996; **275**(3): 189-93.
22. Anell A, Glenngard AH, Merkur S. Sweden health system review. *Health Syst Transit* 2012; **14**(5): 1-159.
23. Socialstyrelsen. Internationell statistisk klassifikation av sjukdomar och relaterade hälsoproblem – Systematisk förteckning, svensk version 2020 (ICD-10-SE). Stockholm: Socialstyrelsen; 2020. Artikelnummer 2020-2-6570.
24. Swedish Board of Health and Welfare [Socialstyrelsen]. *The National Patient Register [Patientregistret]*;2020. https://www.socialstyrelsen.se/en/statistics-and-data/registers/register-information/the-national-patient-register/ [accessed 2020 Feb 8].

25. Ludvigsson JF, Andersson E, Ekbom A, et al. External review and validation of the Swedish national inpatient register. *BMC Public Health* 2011; **11**: 450.
26. Statistical Database, In-patient Care Diagnoses [Internet]. Stockholm: The National Board of Health and Welfare (swe: Socialstyrelsen); 1998-. [cited 2020 Feb 8]. Available from: https://sdb.socialstyrelsen.se/if_par/val_eng.aspx
27. Socialstyrelsen. Anvisningar för val av huvud-och bidiagnos, version 4.1 Stockholm: Socialstyrelsen; 2020. Artikelnummer 2016-05-11.
28. Swedish Board of Health and Welfare [Socialstyrelsen]. *Coding quality of the National Inpatient Register [Kodningskvalitet I patientregistret];2013-3-10.* https://www.socialstyrelsen.se/globalassets/sharepoint-dokument/artikelkatalog/statistik/2013-3-10.pdf [accessed 10 January 2021]
29. European Centre for Disease Prevention and Control. Antimicrobial consumption database (ESAC-Net) [internet]. Stockholm: ECDC; 2020. Available from: https://www.ecdc.europa.eu/en/antimicrobial-consumption/surveillance-and-disease-data/database
30. Organisation for economic cooperation and development (OECD), Health at a Glance 2019: OECD Indicators, OECD Publishing, Paris, https://doi.org/10.1787/4dd50c09-en.
31. Wilson D, Bhopal R. Impact of infection on mortality and hospitalization in the North East of England. *J Public Health Med* 1998; **20**(4): 386-95.
32. Tsao SW, Yip YL, Tsang CM, et al. Etiological factors of nasopharyngeal carcinoma. *Oral Oncol* 2014; **50**(5): 330-8.
33. Cheng XJ, Lin JC, Tu SP. Etiology and Prevention of Gastric Cancer. *Gastrointest Tumors* 2016; **3**(1): 25-36.
34. Clark MA, Hartley A, Geh JI. Cancer of the anal canal. *Lancet Oncol* 2004; **5**(3): 149-57.
35. Villanueva A. Hepatocellular Carcinoma. *N Engl J Med* 2019; **380**(15): 1450-62.
36. Cesarman E, Damania B, Krown SE, Martin J, Bower M, Whitby D. Kaposi sarcoma. *Nat Rev Dis Primers* 2019; **5**(1): 9.
37. Crosbie EJ, Einstein MH, Franceschi S, Kitchener HC. Human papillomavirus and cervical cancer. *Lancet* 2013; **382**(9895): 889-99.
38. Willison HJ, Jacobs BC, van Doorn PA. Guillain-Barré syndrome. *Lancet* 2016; **388**(10045): 717-27.
39. Mills R, Hathorn I. Aetiology and pathology of otitis media with effusion in adult life. *J Laryngol Otol* 2016; **130**(5): 418-24.
40. Rosenfeld RM, Shin JJ, Schwartz SR, et al. Clinical Practice Guideline: Otitis Media with Effusion (Update). *Otolaryngol Head Neck Surg* 2016; **154**(1 Suppl): S1-s41.
41. Sproat R, Burgess C, Lancaster T, Martinez-Devesa P. Eustachian tube dysfunction in adults. *Bmj* 2014; **348**: g1647.
42. Karthikeyan G, Guilherme L. Acute rheumatic fever. *Lancet* 2018; **392**(10142): 161-74.
43. Marijon E, Mirabel M, Celermajer DS, Jouven X. Rheumatic heart disease. *Lancet* 2012; **379**(9819): 953-64.
44. Helbling R, Conficconi E, Wyttenbach M, et al. Acute Nonspecific Mesenteric Lymphadenitis: More Than "No Need for Surgery". *Biomed Res Int* 2017; **2017**: 9784565.
45. Rudmik L, Soler ZM. Medical Therapies for Adult Chronic Sinusitis: A Systematic Review. *Jama* 2015; **314**(9): 926-39.
46. Wood JM, Athanasiadis T, Allen J. Laryngitis. *Bmj* 2014; **349**: g5827.
47. Rabe KF, Watz H. Chronic obstructive pulmonary disease. *Lancet* 2017; **389**(10082): 1931-40.
48. Barker AF. Bronchiectasis. *N Engl J Med* 2002; **346**(18): 1383-93.
49. Kinane DF, Stathopoulou PG, Papapanou PN. Periodontal diseases. *Nat Rev Dis Primers* 2017; **3**: 17038.
50. Robertson DP, Keys W, Rautemaa-Richardson R, Burns R, Smith AJ. Management of severe acute dental infections. *Bmj* 2015; **350**: h1300.
51. Sipponen P, Maaroos HI. Chronic gastritis. *Scand J Gastroenterol* 2015; **50**(6): 657-67.
52. Flum DR. Clinical practice. Acute appendicitis--appendectomy or the "antibiotics first" strategy. *N Engl J Med* 2015; **372**(20): 1937-43.
53. González-Pinto I, González EM. Optimising the treatment of upper gastrointestinal fistulae. *Gut* 2001; **49 Suppl 4**(Suppl 4): iv22-31.
54. Lucchino B, Spinelli FR, Perricone C, Valesini G, Di Franco M. Reactive arthritis: current treatment challenges and future perspectives. *Clin Exp Rheumatol* 2019; **37**(6): 1065-76.
55. Chadban SJ, Atkins RC. Glomerulonephritis. *Lancet* 2005; **365**(9473): 1797-806.
56. Polackwich AS, Shoskes DA. Chronic prostatitis/chronic pelvic pain syndrome: a review of evaluation and therapy. *Prostate Cancer Prostatic Dis* 2016; **19**(2): 132-8.

57. Schaeffer AJ. Clinical practice. Chronic prostatitis and the chronic pelvic pain syndrome. *N Engl J Med* 2006; **355**(16): 1690-8.
58. Caggiano V, Weiss RV, Rickert TS, Linde-Zwirble WT. Incidence, cost, and mortality of neutropenia hospitalization associated with chemotherapy. *Cancer* 2005; **103**(9): 1916-24.
59. Kuderer NM, Dale DC, Crawford J, Cosler LE, Lyman GH. Mortality, morbidity, and cost associated with febrile neutropenia in adult cancer patients. *Cancer* 2006; **106**(10): 2258-66.
60. Neutropen feber – primär handläggning. https://www.internetmedicin.se/page.aspx?id=341 (accessed Feb 9, 2020).
61. Pearce EN, Farwell AP, Braverman LE. Thyroiditis. *N Engl J Med* 2003; **348**(26): 2646-55.
62. Collier SA, Gronostaj MP, MacGurn AK, et al. Estimated burden of keratitis--United States, 2010. *MMWR Morb Mortal Wkly Rep* 2014; **63**(45): 1027-30.
63. Prasad HK, Sreedharan S, Prasad HS, Meyyappan MH, Harsha KS. Perichondritis of the auricle and its management. *J Laryngol Otol* 2007; **121**(6): 530-4.
64. Sapey E, Stockley RA. COPD exacerbations . 2: aetiology. *Thorax* 2006; **61**(3): 250-8.
65. Wedzicha JA, Seemungal TA. COPD exacerbations: defining their cause and prevention. *Lancet* 2007; **370**(9589): 786-96.
66. Mandell LA, Niederman MS. Aspiration Pneumonia. *N Engl J Med* 2019; **380**(7): 651-63.
67. Lanspa MJ, Jones BE, Brown SM, Dean NC. Mortality, morbidity, and disease severity of patients with aspiration pneumonia. *J Hosp Med* 2013; **8**(2): 83-90.
68. Wu CP, Chen YW, Wang MJ, Pinelis E. National Trends in Admission for Aspiration Pneumonia in the United States, 2002-2012. *Ann Am Thorac Soc* 2017; **14**(6): 874-9.
69. Siqueira JF, Jr., Rôças IN. Microbiology and treatment of acute apical abscesses. *Clin Microbiol Rev* 2013; **26**(2): 255-73.
70. Hedlund J, Strålin K, Ortqvist A, Holmberg H. Swedish guidelines for the management of community-acquired pneumonia in immunocompetent adults. *Scand J Infect Dis* 2005; **37**(11-12): 791-805.
71. Ljungström LR, Steinum O, Brink M, Gårdlund B, Martner J, Sjölin J. [Diagnosis and diagnostic coding of severe sepsis and septic shock. ICD-10 should be completed with additional codes]. *Lakartidningen* 2011; **108**(6): 276-8.
